# Comprehensive assessment of novel cardiovascular biomarkers in AF

**DOI:** 10.1093/europace/euag096

**Published:** 2026-05-15

**Authors:** Amelie H Ohlrogge, Daniel Engler, Patricia Schlieker, Ferdinand Seum, Kim Rosebrock, Nicole Nebel, Dora Csengeri, Larissa Fabritz, André Ziegler, Stefan Blankenberg, Paulus Kirchhof, Tanja Zeller, Renate B Schnabel

**Affiliations:** Department of Cardiology, University Heart and Vascular Center Hamburg, Martinistrasse 52, 20251 Hamburg, Germany; German Center for Cardiovascular Research (DZHK), Partner Site North, Martinistrasse 52, 20251 Hamburg, Germany; Department of Cardiology, University Heart and Vascular Center Hamburg, Martinistrasse 52, 20251 Hamburg, Germany; German Center for Cardiovascular Research (DZHK), Partner Site North, Martinistrasse 52, 20251 Hamburg, Germany; Department of Cardiology, University Heart and Vascular Center Hamburg, Martinistrasse 52, 20251 Hamburg, Germany; German Center for Cardiovascular Research (DZHK), Partner Site North, Martinistrasse 52, 20251 Hamburg, Germany; Department of Cardiology, University Heart and Vascular Center Hamburg, Martinistrasse 52, 20251 Hamburg, Germany; German Center for Cardiovascular Research (DZHK), Partner Site North, Martinistrasse 52, 20251 Hamburg, Germany; Department of Cardiology, University Heart and Vascular Center Hamburg, Martinistrasse 52, 20251 Hamburg, Germany; German Center for Cardiovascular Research (DZHK), Partner Site North, Martinistrasse 52, 20251 Hamburg, Germany; Department of Cardiology, University Heart and Vascular Center Hamburg, Martinistrasse 52, 20251 Hamburg, Germany; Department of Cardiology, University Heart and Vascular Center Hamburg, Martinistrasse 52, 20251 Hamburg, Germany; German Center for Cardiovascular Research (DZHK), Partner Site North, Martinistrasse 52, 20251 Hamburg, Germany; Department of Cardiology, University Heart and Vascular Center Hamburg, Martinistrasse 52, 20251 Hamburg, Germany; German Center for Cardiovascular Research (DZHK), Partner Site North, Martinistrasse 52, 20251 Hamburg, Germany; University Center of Cardiovascular Science, University Heart and Vascular Center Hamburg, University Medical Center Hamburg Eppendorf, Hamburg, Germany; Institute of Cardiovascular Sciences, University of Birmingham, Birmingham, UK; Roche Diagnostics International Ltd, Rotkreuz, Switzerland; Department of Cardiology, University Heart and Vascular Center Hamburg, Martinistrasse 52, 20251 Hamburg, Germany; German Center for Cardiovascular Research (DZHK), Partner Site North, Martinistrasse 52, 20251 Hamburg, Germany; Department of Cardiology, University Heart and Vascular Center Hamburg, Martinistrasse 52, 20251 Hamburg, Germany; German Center for Cardiovascular Research (DZHK), Partner Site North, Martinistrasse 52, 20251 Hamburg, Germany; Institute of Cardiovascular Sciences, University of Birmingham, Birmingham, UK; University Hospital Schleswig-Holstein Lübeck, University of Lübeck, Institute for Cardiogenetics, Lübeck, Germany; German Center for Cardiovascular Research (DZHK), Partner Site North, Lübeck, Germany; Department of Cardiology, University Heart and Vascular Center Hamburg, Martinistrasse 52, 20251 Hamburg, Germany; German Center for Cardiovascular Research (DZHK), Partner Site North, Martinistrasse 52, 20251 Hamburg, Germany

**Keywords:** Atrial fibrillation, Biomarkers, Angiopoietin 2, Bone morphogenetic protein 10, Fibroblast growth factor 23, Insulin-like growth factor binding protein 7, N-terminal pro B-type natriuretic peptide

## Abstract

**Aims:**

Biomarkers have the potential to improve risk prediction beyond clinical characteristics. We examined the association of four emerging cardiovascular biomarkers [angiopoietin 2 (Angpt2), bone morphogenetic protein 10 (BMP10), fibroblast growth factor 23 (FGF23), insulin-like growth factor binding protein 7 (IGFBP7)] in comparison with N-terminal pro B-type natriuretic peptide (NT-proBNP) across the disease course of atrial fibrillation (AF).

**Methods and results:**

We enrolled patients from a prospective cohort of patients at risk of AF or with manifest arrhythmia. The circulating vascular biomarkers were quantified using high-throughput, high-precision precommercial assays (Roche Diagnostics). A combined endpoint comprised: stroke, transient ischaemic attack (TIA), myocardial infarction, incident coronary heart disease, heart failure, and all-cause mortality. Of the total *n* = 1047 individuals, *n* = 527 had prevalent AF, and *n* = 507 were free of AF at baseline. Median follow-up was 42 months. A total of *n* = 66 individuals died; the combined endpoint occurred in *n* = 198 individuals. All five biomarkers were significantly associated with the incidence of AF, both in multistate analysis (MSA) and Cox regression, although the association with FGF23 was only significant in the age- and sex-adjusted Cox model. AF recurrence was significantly associated with all biomarkers, most strongly with NT-proBNP. In prevalent AF, NT-proBNP, FGF23, and IGFBP7 were associated with the combined endpoint and all-cause mortality, and Angpt2 was associated with all-cause mortality. NT-proBNP showed the strongest association for all-cause mortality, and IGFBP7 for the combined endpoint in prevalent AF. In incident AF the association with the combined outcome was statistically significant for NT-proBNP in multivariable-adjusted models. All-cause mortality in individuals with incident AF was associated with NT-proBNP, Angpt2, FGF23, and IGFBP7 both in the MSA and Cox model.

**Conclusion:**

All novel biomarkers Angpt2, BMP10, FGF23, and IGFBP7 showed predictive value for incident and recurrent AF. Individual biomarkers showed distinct strengths in prediction of outcomes across the disease spectrum of AF.

## Graphical Abstract

**Graphical Abstract:**
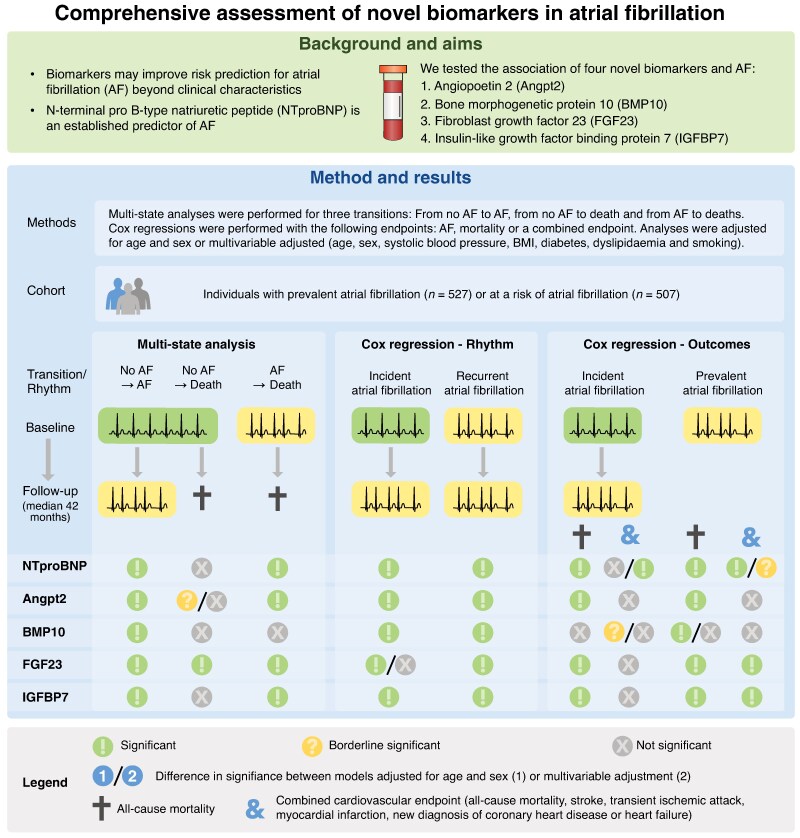
Abbreviations: Angpt2—angiopoietin 2, BMP10—bone morphogenetic protein 10, FGF 23—fibroblast growth factor 23, HR—hazard ratio, IGFBP7—insulin-like growth factor binding protein 7, NT-proBNP—N-terminal pro B-type natriuretic peptide. Analyses are explorative.

What’s new?

- In a prospective cohort of over 1,000 individuals across the entire spectrum of atrial fibrillation (AF) risk, four novel biomarkers — angiopoietin 2 (Anpt2), bone morphogenetic protein 10 (BMP10), fibroblast growth factor 23 (FGF23), and insulin-like growth factor binding protein 7 (IGFBP7) — were associated with incident and recurrent AF in both multi-state analysis and Cox regressions, extending prior evidence from selected AF populations, e.g. post-ablation.
- Individual biomarkers showed distinct predictive strengths across the AF disease course: Angpt2 and NT-proBNP had the strongest association with incident AF and mortality, and IGFBP7 was strongly associated with combined cardiovascular outcomes in prevalent AF.

## Introduction

Atrial fibrillation (AF) is the most prevalent arrhythmia associated with increased morbidity and mortality.^1,2^ The risk prediction of a first AF event, recurrent AF events, complications such as a stroke or death remains challenging. Basic clinical features such as age, comorbidity, and sex are the cornerstones to predict the risk of incident AF or AF complications. The addition of biomarkers to clinical parameters can improve risk prediction. A well-established biomarker in relation to AF risk is N-terminal pro B-type natriuretic peptide (NT-proBNP).^3^ Novel candidate biomarkers have been identified with potential to improve risk prediction in AF: angiopoietin 2 (Angpt2), bone morphogenetic protein 10 (BMP10), fibroblast growth factor 23 (FGF23), and insulin-like growth factor binding protein 7 (IGFBP7). These have been shown to indicate the presence of AF,^4^ its risk of cardiovascular complications and all-cause death,^5–7^ and AF recurrence.^8–10^

The four emerging biomarkers examined in this study are involved in cardiovascular remodelling via a complex interplay of different regulatory pathways. Angpt2 is a growth factor which is involved in angiogenesis and vascular remodelling, and its expression is triggered by inflammatory mediators.^11^ It has been demonstrated that Angpt2 is involved in inflammatory atrial fibrosis.^12^ BMP10 modulates processes in the cardiovascular system, among others, cardiac repair and cardiomyocyte proliferation.^13^ BMP10 is secreted by human atrial myocytes and is significantly increased in individuals with AF.^14^ A recent Mendelian randomization study has suggested non-causal associations between BMP10 and AF.^15^ FGF23 is a hormone mainly involved in phosphate homeostasis and active vitamin D regulation.^16^ It regulates atrial fibrosis in AF and calcium signalling.^17,18^ Some IGFBP7 pathways have been related to the senescence of cardiomyocytes and pathological cardiac remodelling.^19^

We aimed to test the associations and discriminatory ability of these novel biomarkers in comparison in a prospective outpatient cohort including a broad range of individuals across the spectrum of AF, i.e. in patients at risk of developing the disease and in prevalent AF. We further compared their performance to the routine biomarker NT-proBNP.

## Methods

### Study cohort

The Atrial Fibrillation in High-Risk Individuals (AFHRI) study is an ongoing, prospective cohort study designed to improve AF risk prediction. AFHRI is a sub-study of the clinical cohort study (CCS) conducted at the University Heart and Vascular Centre Hamburg, Germany. We included patients aged 18 years or older at risk of incident or recurrent AF across the whole spectrum of risk. Patients were recruited between 1 December 2012 and 30 June 2021. Individuals who did not have sufficient knowledge of the German language to understand the informed consent forms and to participate in the interview were excluded.

### Data collection

Baseline data were collected by physical examination, an interview using a detailed questionnaire, including information on pre-existing conditions, medication, family history, lifestyle, and cardiovascular risk factors. Baseline information was supplemented by a review of the electronic medical record. For each study participant, all electrocardiograms were analysed by two experienced investigators. In the case of discrepancies in the diagnosis of AF, a third cardiologist or electrophysiologist was consulted. Heart failure classification was based on a combination of clinical signs (dyspnoea or oedema), diuretic heart failure medication (loop diuretics or aldosterone receptor antagonists), echocardiography, and NT-proBNP plasma concentrations in accordance with the most recent 2021 ESC heart failure guidelines.^20^

### Biomarker measurements

Blood was drawn, processed within 30 min, and stored at −80°C. Absolute protein concentrations were quantified in EDTA plasma using standardized procedures. NT-proBNP plasma concentrations were determined in-house at the study site using the Elecsys 2010 platform (ECLIA, Roche Diagnostics) via an electrochemiluminescence immunoassay. The analytical range was 5–35 000 pg/mL, intra- and interassay variation coefficients of variation were 1.38 and 2.58%, respectively.

The experimental biomarkers were determined at Roche using a cobas e601 analyser (Roche Diagnostics, Penzberg, Germany) using non-commercial, robust prototype Elecsys electrochemiluminescence immunoassays (Roche Diagnostics, Penzberg, Germany). For Angpt2, the intra- and interassay coefficients of variation were 5.2 and 4.0% at concentrations of 0.98 and 3.40 ng/mL, respectively. For BMP10 the lower detection limit was 0.003 ng/mL, with a functional sensitivity (lower limit of quantification) of 0.012 ng/mL, an upper measuring range of 10 ng/mL, and a coefficient of variation of 2.35% (mean: 1.38 ng/mL). FGF23 plasma concentrations were measured using a rabbit monoclonal antibody-based immunoassay with a functional sensitivity of 4 pg/mL and a within-run coefficient of variation of 1.7%. IGFBP7 concentrations were measured with a precision within-run coefficient variation of 2% and a lower detection limit of 0.01 ng/mL. All biomarker analyses were conducted under stringent quality control and calibration protocols by laboratory personnel, who were blinded to clinical data.

### Statistical analysis

Continuous variables are presented as median (25th, 75th percentile), and categorical variables are presented as frequencies and percentages. Biomarker measurement outliers were identified by graphical representations in boxplots and scatterplots. In addition, we considered a *Z*-score of +/−2.6 or more as an outlier. Outliers were excluded from analysis after the additional check of distribution plots. We log-transformed biomarkers for regression analyses.

Binary logistic regression models and Cox regressions were used to assess the associations of these biomarkers for group differences. We calculated two models: (i) an age- and sex-adjusted (ii) model and a model additionally adjusted for systolic blood pressure, body mass index (BMI), diabetes, dyslipidaemia, and smoking. In a sensitivity analysis, we additionally adjusted for heart failure at baseline. Furthermore, we included the glomerular filtration rate (eGFR) as an additional covariate in the models. As 7.5% of the observations for this variable were missing, multiple imputation was applied to handle missing data. The imputation procedure was performed using 20 iterations to generate plausible values for the missing observations. Model performance was assessed using the area under the receiver operating characteristic curve (ROC-AUC) to evaluate discrimination, and the adjusted coefficient of determination (adjusted *R*^2^) to assess model fit. The optimal classification threshold was determined using Youden’s index (sensitivity + specificity − 1).

The correlation between nominal variables and interval-scaled variables was calculated using the Pearson correlation coefficient. All statistical models were conducted using SPSS 29.0 (SPSS Inc., Chicago, IL, USA). The transition models were conducted using R 4.5.2 (R Foundation for Statistical Computing, Vienna, Austria, Version 2025.12.1). A two-tailed *P*-value <0.05 was considered statistically significant, and confidence intervals were estimated at 95%.

In addition to these analyses, we implemented a semi-parametric, transition-specific Cox-type multistate model to describe the event process from the AF-free state through the onset of AF to all-cause mortality. The model was specified as a three-state illness–death model with the following states: no AF (initial state), AF (intermediate state), and death (absorbing state). All individuals entered the model in the no AF state. We defined three possible transitions: (i) transition from no AF to AF, (ii) transition from AF to death, and (iii) transition from no AF to death. The local ethics committee approved the observational study (PV5705/PV3982). Participation in the study was voluntary. Participants signed written informed consent. The authors had full access to the data and take responsibility for its integrity. All authors have read and agreed to the manuscript as written.

## Results

### Baseline characteristics

In total 1047 individuals were included in the analysis, and 36.0% were women. The median age was lowest in those without AF during follow-up and highest in those with incident AF. The other risk factors and comorbidities did not differ significantly between groups. The biomarkers NT-proBNP and IGFBP7 were significantly higher in the incident AF group compared to those without AF (*P* < 0.001 and *P* = 0.018, respectively). Median follow-up was 42 months. During follow-up significantly more individuals with incident AF died compared to those without AF (18.1 and 7.4%, *P* = 0.002) or suffered from myocardial infarction or angina pectoris (11.1 and 4.0%, *P* = 0.006). The incidence of stroke/transient ischaemic attack (TIA) or coronary heart disease did not differ significantly (*Table 1*).

**Table 1:** Baseline characteristics of patients at risk of atrial fibrillation by disease state

Variables	No AF	Prevalent AF	Incident AF
*n*, (%)	464 (44.9)	527 (51.0)	43 (4.2)
Risk factors			
Age, years	58.2 (49.0, 69.7)	64.8 (50.8, 72.7)	67.3 (56.8, 71.8)
Women, *n* (%)	206 (44.4)	153 (29.0)	13 (30.2)
Systolic BP, mmHg	135 (120, 149)	133 (120, 145)	137 (120, 145)
Diastolic BP, mmHg	80 (71, 85)	80 (70, 85)	80 (70, 84)
BMI, kg/m^2^	25.8 (23.6, 29.3)	26.5 (24.2, 29.4)	26.8 (24.6, 29.7)
Obesity, *n* (%)	103 (22.2)	114 (21.6)	9 (20.9)
Current smoking, *n* (%)	107 (23.1)	62 (11.8)	7 (16.3)
Former smoking, *n* (%)	184 (39.7)	219 (41.6)	17 (39.5)
eGFR, mL/min/1,73 m^2^	88 (73, 99)	82 (68, 94)	87 (66, 95)
AF Type			
Paroxysmal	—	249 (47.3)	—
Persistent	—	13 (2.5)	—
Permanent	—	264 (50.2)	—
Rhythm at study inclusion			
Sinus rhythm	464 (100)	382 (73.3)	43 (100)
Atrial fibrillation		118 (22.6)	
Atrial flutter		14 (2.7)	
Other rhythm		7 (1.3)	
Biomarkers			
NT-proBNP, pg/mL	95 (41, 245)	167 (69, 474)	256 (133, 556)
Angpt2, ng/mL	1.43 (1.16, 1.93)	1.77 (1.30, 2.56)	1.54 (1.25, 2.44)
BMP10, ng/mL	1.92 (1.70, 2.25)	2.10 (1.83, 2.39)	2.17 (1.87, 2.40)
FGF23, pg/mL	127 (101, 176)	138 (108, 198)	147 (117, 227)
IGFBP7, ng/mL	82 (73, 95)	89 (78, 104)	90 (77, 112)
Comorbidities			
CHA_2_DS_2_-VA score	3 (2, 4)	2 (1, 4)	3 (3, 4)
Heart dailure, *n* (%)	216 (46.6)	300 (56.9)	22 (51.2)
HFpEF, *n* (%)	152 (70.4)	202 (38.3)	15 (68.2)
HFmrEF, *n* (%)	34 (15.7)	54 (10.2)	4 (18.2)
HFrEF, *n* (%)	30 (6.5)	44 (8.3)	3 (13.6)
Diabetes mellitus, *n* (%)	61 (13.1)	60 (11.4)	10 (23.3)
Hypertension, *n* (%)	337 (72.6)	367 (69.6)	36 (83.7)
Dyslipidaemia, *n* (%)	255 (55.0)	216 (41.1)	27 (62.8)
Stroke/TIA, *n* (%)	177 (38.1)	50 (9.5)	12 (27.9)
Myocardial infarction, *n* (%)	77 (16.6)	36 (6.8)	9 (20.9)
Coronary artery diseases, *n* (%)	120 (25.9)	82 (15.6)	18 (41.9)
Follow-up			
All-cause mortality, *n* (%)	29 (6.3)	28 (5.3)	8 (18.6)
Stroke/TIA, *n* (%)	24 (5.2)	18 (3.4)	2 (4.7)
Myocardial infarction/angina pectoris, *n* (%)	13 (2.8)	12 (2.3)	6 (14.0)
Coronary artery disease, *n* (%)	7 (1.9)	9 (1.7)	2 (4.7)
*Combined outcome* All-cause mortality, stroke/TIA, myocardial infarction, coronary artery disease	87 (18.8)	90 (17.1)	19 (44.2)

Data are presented as *n* (percentages) for categorical variables and as median (1st quartile, 3rd quartile) for continuous variables.

Abbreviations: Angpt2, angiopoietin 2; BMP10, bone morphogenetic protein 10; CHA_2_DS_2_-VA score, congestive heart failure (1), hypertension (1), age ≥75 years (2), diabetes mellitus (1), stroke/TIA (1), vascular disease (1), age 65–74 years (1); FGF23, fibroblast growth factor 23; HFmrEF, heart failure with mildly reduced ejection fraction; HFrEF, heart failure with reduced ejection fraction; HFpEF, heart failure with preserved ejection fraction; IGFBP7, insulin-like growth factor binding protein 7; NT-proBNP, N-terminal pro B-natriuretic peptide; TIA, transient ischaemic attack.

### Correlation of biomarkers

All five log-transformed biomarkers correlated with each other, with correlation coefficients *r* ranging from 0.218 (FGF23 and NT-proBNP) to 0.452 (Angpt2 and NT-proBNP) (see Supplementary material online, *Figure S1*). Age correlated most strongly with the biomarkers, with *r* ranging from 0.166 (FGF23) to 0.439 (NT-proBNP). Creatinine correlated most strongly with IGFBP7 (*r* = 0.409)and less with BMP10 (*r* = 0.063).

### Multistate analysis

#### Transition from no atrial fibrillation to atrial fibrillation

**Figure 1:**
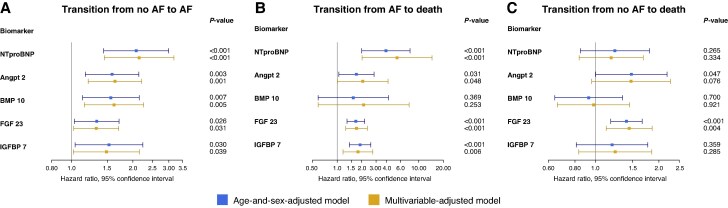
Forest plots showing hazard ratios for the transition from no AF to AF (*A*), transition from AF to death (*B*), and transition from no AF to death (*C*). The age- and sex-adjusted models are shown in blue and the multivariable-adjusted models in yellow. Adjustment was performed for age, sex, systolic blood pressure, BMI, diabetes, dyslipidaemia, and smoking. Abbreviations: AF, atrial fibrillation; Angpt2, angiopoietin 2; BMP10, bone morphogenetic protein 10; FGF23, fibroblast growth factor 23; IGFBP7, insulin-like growth factor binding protein 7.

In multistate analysis (MSA) of individuals without AF at baseline, all five biomarkers were significantly associated with the transition to AF in both the age-and-sex- and multivariable-adjusted models (*Table 2*, *Figure 1*, Panel A). The strongest association was observed for NT-proBNP (HR 2.15, 95% CI 1.45–3.18 multivariable-adjusted).

**Table 2:** Hazard ratios for the transition from no AF to AF in the MSA

Biomarkers	HR	95% confidence interval	
		Lower	Upper
Adjusted for age and sex			
NT-proBNP	2.08	1.44	2.99
Angpt2	1.58	1.17	2.14
BMP10	1.56	1.13	2.15
FGF23	1.33	1.03	1.71
IGFBP7	1.53	1.04	2.24
Multivariable adjustment			
NT-proBNP	2.15	1.45	3.18
Angpt2	1.63	1.21	2.21
BMP10	1.62	1.16	2.25
FGF23	1.32	1.03	1.70
IGFBP7	1.48	1.02	2.15

Multivariable-adjusted model: age, sex, systolic blood pressure, BMI, diabetes, dyslipidaemia, and smoking. Statistically significant observations at the 0.05 threshold are marked in bold.

Abbreviations: Angpt2, angiopoietin 2; BMP10, bone morphogenetic protein 10; FGF23, fibroblast growth factor 23; IGFBP7, insulin-like growth factor binding protein 7; NT-proBNP, N-terminal pro brain-natriuretic peptide.

#### Transition from atrial fibrillation to death

The transition from AF to death was associated with Angpt2, FGF23, IGFBP7, and NT-proBNP in both models, most strongly for NT-proBNP (HR 5.42, 95% CI 2.02–14.55 multivariable-adjusted) (*Table 3*, *Figure 1*, Panel B). BMP10 was not statistically significantly associated with the transition from AF to death.

**Table 3:** Hazard ratios for the transition from AF to death in the MSA

Biomarkers	HR	95% confidence interval	
		Lower	Upper
Adjusted for age and sex			
NT-proBNP	**3**.**96**	**2**.**00**	**7**.**81**
Angpt2	**1**.**72**	**1**.**05**	**2**.**83**
BMP10	1.57	0.58	4.24
FGF23	**1**.**69**	**1**.**32**	**2**.**17**
IGFBP7	**1**.**91**	**1**.**41**	**2**.**59**
Multivariable adjustment			
NT-proBNP	**5**.**42**	**2**.**02**	**14**.**55**
Angpt2	**2**.**06**	**1**.**01**	**4**.**21**
BMP10	2.11	0.59	7.61
FGF23	**1**.**73**	**1**.**27**	**2**.**34**
IGFBP7	**1**.**80**	**1**.**18**	**2**.**74**

Multivariable-adjusted model: age, sex, systolic blood pressure, BMI, diabetes, dyslipidaemia, and smoking. Statistically significant observations at the 0.05 threshold are marked in bold.

Abbreviations: Angpt2, angiopoietin 2; BMP10, bone morphogenetic protein 10; FGF23, fibroblast growth factor 23; IGFBP7, insulin-like growth factor binding protein 7; NT-proBNP, N-terminal pro brain-natriuretic peptide.

#### Transition from no atrial fibrillation to death

The transition to death without interim AF was associated with FGF23 (HR 1.45, 95% CI 1.13–1.86 multivariable-adjusted) in both models, and showed a borderline association with Angpt2 in the age-and-sex-adjusted model (HR 1.48, 95% CI 1.00–2.19) (*Table 4*, *Figure 1*, Panel C). None of the other biomarkers were associated with the transition to death without interim AF.

**Table 4:** Hazard ratios for the transition from no AF to AF in the MSA

Biomarkers	HR	95% confidence interval	
		Lower	Upper
Adjusted for age and sex			
NT-proBNP	1.24	0.85	1.80
Angpt2	**1**.**48**	**1**.**00**	**2**.**19**
BMP10	0.93	0.64	1.35
FGF23	**1**.**40**	**1**.**18**	**1**.**67**
IGFBP7	1.20	0.81	1.77
Multivariable adjustment			
NT-proBNP	1.19	0.84	1.69
Angpt2	1.48	0.96	2.27
BMP10	0.98	0.66	1.45
FGF23	**1**.**45**	**1**.**13**	**1**.**86**
IGFBP7	1.24	0.83	1.85

Multivariable-adjusted model: age, sex, systolic blood pressure, BMI, diabetes, dyslipidaemia, and smoking. Statistically significant observations are marked in bold.

Abbreviations: Angpt2, angiopoietin 2; BMP10, bone morphogenetic protein 10; FGF23, fibroblast growth factor 23; IGFBP7, insulin-like growth factor binding protein 7; NT-proBNP, N-terminal pro brain-natriuretic peptide.

### Cox regressions across outcomes

#### Incident atrial fibrillation

**Figure 2:**
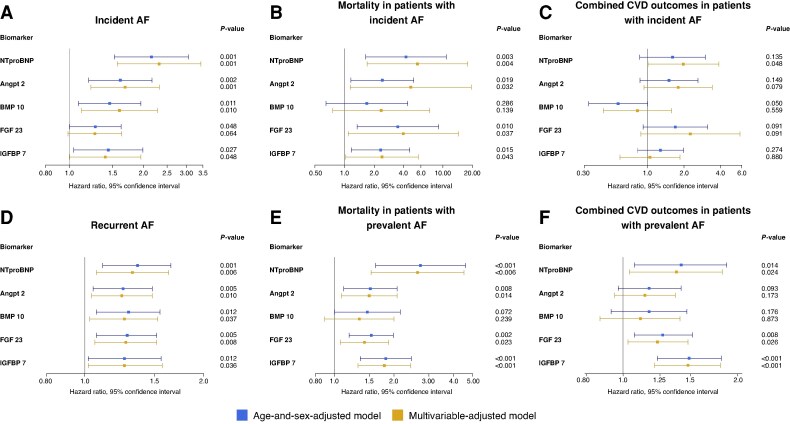
Forest plots showing hazard ratios and 95% confidence intervals for incident AF (*A*), all-cause mortality in patients with incident AF (*B*), combined CVD outcomes in patients with incident AF (*C*), recurrent AF (*D*), all-cause mortality in patients with prevalent AF (*E*) and combined CVD outcomes in patients with prevalent AF (*F*). The age- and sex-adjusted models are shown in blue and the multivariable-adjusted models in yellow. Abbreviations: AF, atrial fibrillation; Angpt2, angiopoietin 2; BMP10, bone morphogenetic protein 10; CVD, cardiovascular disease; FGF23, fibroblast growth factor 23; IGFBP7, insulin-like growth factor binding protein 7.

In 502 patients free of AF at baseline, all biomarkers were associated with incident AF in age- and sex-adjusted analyses. The strongest associations were observed for NT-proBNP and Angpt2 (HR 2.16, 95% CI 1.53–3.05, *P* < 0.001, and HR 1.61, 95% CI 1.20–2.17, *P* = 0.002, respectively) (*Table 5*, *Figure 2*, Panel A). After multivariable adjustment this association remained statistically significant for all biomarkers, except for FGF23.

**Table 5:** Cox regressions for log-transformed biomarker concentrations in relation to incident AF

Biomarkers (*n*)^a^	HR	95% confidence interval		*P*-value
		Lower	Upper	
Adjusted for age and sex				
NT-proBNP (498)	2.16	1.53	3.05	**<0**.**001**
Angpt2 (499)	1.61	1.20	2.17	**0**.**002**
BMP10 (502)	1.46	1.09	1.96	**0**.**011**
FGF23 (502)	1.28	1.00	1.62	**0**.**048**
IGFBP7 (502)	1.44	1.04	1.99	**0**.**027**
Multivariable adjustment				
NT-proBNP (486)	2.32	1.57	3.43	**<0**.**001**
Angpt2 (488)	1.69	1.22	2.33	**0**.**001**
BMP10 (490)	1.60	1.12	2.29	**0**.**010**
FGF23 (490)	1.27	0.99	1.63	0.064
IGFBP7 (490)	1.40	1.00	1.96	**0**.**048**

Statistically significant regressions (*P* < 0.05) are marked in bold. Analyses are based on 42 events.

Abbreviations: Angpt2, angiopoietin 2; BMP10, bone morphogenetic protein 10; FGF23, fibroblast growth factor 23; IGFBP7, insulin-like growth factor binding protein 7; NT-proBNP, N-terminal pro brain-natriuretic peptide.

^a^Numbers in parentheses are cases included in the analysis. Multivariable-adjusted model: age, sex, systolic blood pressure, BMI, diabetes, dyslipidaemia, and smoking.

**Figure 3:**
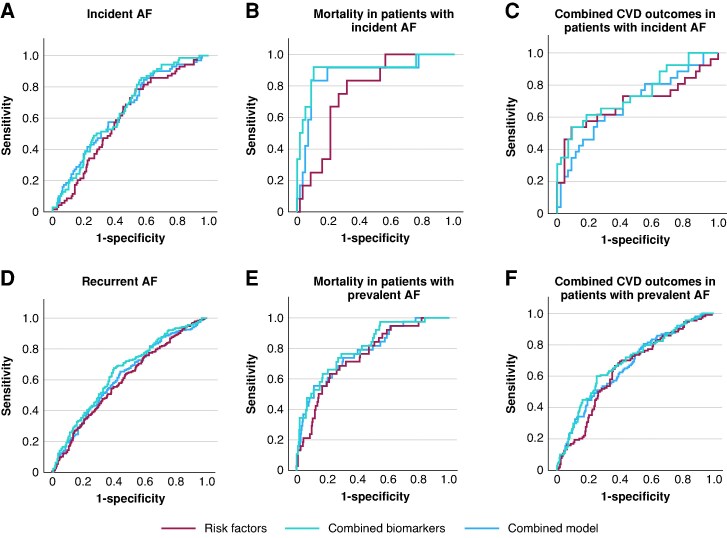
Receiver operating characteristic curve for (i) the combined biomarkers (NT-proBNP, Angpt2, BMP10, FGF23, IGFBP7), (ii) risk factors (age, sex, systolic blood pressure, BMI, diabetes, dyslipidaemia and smoking), and (iii) a combined model of biomarkers and risk factors for ratios for incident AF (*A*), all-cause mortality in patients with incident AF (*B*), combined CVD outcomes in patients with incident AF (*C*), recurrent AF (*D*), all-cause mortality in patients with prevalent AF (*E*), and combined CVD outcomes in patients with prevalent AF (*F*). Abbreviations: AF, atrial fibrillation; Angpt2, angiopoietin 2; BMP10, bone morphogenetic protein 10; CVD, cardiovascular disease; FGF23, fibroblast growth factor 23; IGFBP7, insulin-like growth factor binding protein 7.

The predictive ability of all five biomarkers combined was slightly higher than the predictive ability of the combined risk factors. The difference did not meet statistical significance (AUC 0.643 and 0.606, respectively, *P*-value for difference 0.355). The combined model including risk factors and biomarkers was not statistically significantly superior to either model alone (AUC 0.652, *P*-values for difference 0.618 and 0.098, respectively) (*Figure 3*, Panel A, Supplementary material online, *Table S1*). The receiver operating characteristics (ROC) were highest for NT-proBNP (0.751), followed by Angpt2 (0.731) and BMP10 (0.724) (see Supplementary material online, *Table*  S2 and *Figure S2*).

##### All-cause mortality in patients with incident atrial fibrillation

In both age- and sex-adjusted and multivariable Cox regressions, NT-proBNP, Angpt2, FGF23, and IGFBP7 were significantly associated with all-cause mortality in patients with incident AF. In multivariable-adjusted models, this association was strongest for NT-proBNP (HR 5.52, 95% CI 1.70–17.90, *P* = 0.004), followed by Angpt2 (HR 4.73, 95% CI 1.1.4–19.57, *P* = 0.032) (*Table 6*, *Figure 1*, Panel B).

**Table 6:** Cox regressions for log-transformed biomarkers in relation to all-cause mortality in patients with incident AF

Biomarkers (*n*)^a^	HR	95% confidence interval		*P*-value
		Lower	Upper	
Adjusted for age and sex				
NT-proBNP (39)	4.22	1.65	10.81	**0**.**003**
Angpt2 (39)	2.42	1.16	5.06	**0**.**019**
BMP10 (39)	1.69	0.65	4.40	0.286
FGF 23 (39)	3.49	1.34	9.06	**0**.**010**
IGFBP7 (39)	2.33	1.18	4.62	**0**.**015**
Multivariable adjustment^b^				
NT-proBNP (38)	5.52	1.70	17.90	**0**.**004**
Angpt2 (39)	4.73	1.14	19.57	**0**.**032**
BMP10 (39)	2.36	0.76	7.36	0.139
FGF23 (39)	3.96	1.09	14.44	**0**.**037**
IGFBP7 (39)	2.41	1.03	5.63	**0**.**043**

Statistically significant regressions (*P* < 0.05) marked in bold. Analyses are based on eight events.

Abbreviations: Angpt2, angiopoietin 2; BMP10, bone morphogenetic protein 10; FGF23, fibroblast growth factor 23; IGFBP7, insulin-like growth factor binding protein 7; NT-proBNP, N-terminal pro brain-natriuretic peptide.

^a^Numbers in parentheses are cases included in the analysis.

^b^Model adjusted for age, sex, systolic blood pressure, BMI, diabetes, dyslipidaemia, and smoking. Combined endpoints include: all-cause mortality, stroke/TIA, myocardial infarction, coronary heart disease, and heart failure.

The biomarker and risk factor model did not differ significantly in their predictive abilities, *n* = 13 events. The addition of biomarkers significantly improved the predictive ability of the risk factor model (AUC 0.904 and 0.766, respectively, *P*-value for difference 0.039) (*Figure 2*, Panel B, Supplementary material online, *Table S1*). NT-proBNP had the highest ROC (0.856), followed by IGFBP7 (0.838) and FGF23 (0.816) (see Supplementary material online, *Table S2* and *Figure S3*).

##### Combined cardiovascular disease outcomes in patients with incident atrial fibrillation

NT-proBNP was significantly associated with combined cardiovascular disease (CVD) outcomes in patients with interim AF during follow-up in the multivariable-adjusted model (HR 1.98, 95% CI 1.01–3.88, *P* = 0.048). BMP10 showed a borderline significant protective association in the model adjusted for age and sex (HR 0.57, 95% CI 0.33–1.00, *P* = 0.050) (*Table 7*, *Figure 1*, Panel C).

**Table 7:** Cox regression of log-transformed biomarkers in relation to combined outcomes in patients with incident AF

Biomarkers (*n*)^a^	HR	95% confidence interval		*P*-value
		Lower	Upper	
Adjusted for age and sex				
NT-proBNP (41)	1.61	0.86	3.00	0.135
Angpt2 (42)	1.50	0.87	2.60	0.149
BMP10 (42)	0.57	0.33	1.00	**0**.**050**
FGF23 (42)	1.79	0.92	3.13	0.091
IGFBP7 (42)	1.28	0.82	1.98	0.274
Multivariable adjustment^b^				
NT-proBNP (41)	1.98	1.01	3.88	**0**.**048**
Angpt2 (42)	1.79	0.93	3.43	0.079
BMP10 (42)	0.82	0.43	1.56	0.559
FGF23 (42)	2.26	0.88	5.80	0.091
IGFBP7 (42)	1.05	0.59	1.85	0.880

Statistically significant regression models (*P* < 0.05) are marked in bold. Analyses are based on 22 events. Combined endpoint includes: all-cause mortality, stroke/TIA, myocardial infarction, coronary heart disease, and heart failure.

Abbreviations: Angpt2, angiopoietin 2; BMP10, bone morphogenetic protein 10; FGF23, fibroblast growth factor 23; IGFBP7, insulin-like growth factor binding protein 7; NT-proBNP, N-terminal pro brain-natriuretic peptide.

^a^Numbers in parentheses are cases included in the analysis.

^b^Model adjusted for age, sex, systolic blood pressure, BMI, diabetes, dyslipidaemia, and smoking.

The predictive ability of the biomarker and risk factor model was similar (AUC 0.684 and 0.690, respectively, *P*-value for difference 0.944); the combined model was non-significantly superior to both (AUC 0.744, *P*-values for difference 0.288 and 0.262, respectively) (*Figure 2*, Panel C, Supplementary material online, *Table S1*). FGF23 had the highest ROC (0.721), followed by BMP10 and IGFB7 (both 0.697) (see Supplementary material online, *Table S2* and *Figure S4*).

#### Prevalent atrial fibrillation

##### Recurrent atrial fibrillation

All five biomarkers were significantly associated with recurrent AF in both Cox regression models (*Table 8*, *Figure 1*, Panel D). The association was comparably strong for all biomarkers, with an HR ranging between 1.27 and 1.60 in the age- and sex-adjusted model, and 1.23 and 1.35 in the multivariable-adjusted model. Cox models for all-cause mortality and the combined cardiovascular outcome in individuals with recurrent AF are displayed in Supplementary material online, *Tables S3* and *S4*.

**Table 8:** Cox regressions for log-transformed biomarkers for recurrent AF

Biomarkers (*n*)^a^	HR	95% confidence interval		*P*-value
		Lower	Upper	
Adjusted for age and sex				
NT-proBNP (414)	1.39	1.14	1.69	**0**.**001**
Angpt2 (416)	1.28	1.08	1.51	**0**.**005**
BMP10 (412)	1.27	1.05	1.53	**0**.**012**
FGF 23 (412)	1.28	1.07	1.53	**0**.**005**
IGFBP7 (427)	1.60	1.11	2.303	**0**.**012**
Multivariable adjustment^b^				
NT-proBNP (402)	1.35	1.09	1.67	**0**.**006**
Angpt2 (402)	1.26	1.26	1.50	**0**.**010**
BMP10 (402)	1.23	1.01	1.51	**0**.**037**
FGF23 (400)	1.28	1.07	1.53	**0**.**008**
IGFBP7 (415)	1.26	1.02	1.56	**0**.**036**

Statistically significant regressions (*P* < 0.05) marked in bold. Analyses are based on 143 events.

Abbreviations: Angpt2, angiopoietin 2; BMP10, bone morphogenetic protein 10; FGF23, fibroblast growth factor 23; IGFBP7, insulin-like growth factor binding protein 7; NT-proBNP, N-terminal pro brain-natriuretic peptide.

^a^Numbers in parentheses are cases included in the analysis.

^b^Model adjusted for age, sex, systolic blood pressure, BMI, diabetes, dyslipidaemia, and smoking.

The predictive ability of the biomarker and risk factor models did not differ (AUC 0.616 and 0.625, respectively, *P*-value for difference 0.825). The addition of biomarkers did not improve the predictive ability compared to the risk factor model (AUC 0.666 for the combined model, *P*-value for difference 0.031) (*Figure 2*, Panel D, Supplementary material online, *Table S1*). ROC was highest for FGF23 (0.662), followed by BMP10 (0.645) (see Supplementary material online, *Table S2* and *Figure S5*).

##### All-cause mortality in patients with atrial fibrillation at baseline

All biomarkers except BMP10 were significantly associated with all-cause mortality in patients with AF at baseline, both in age- and sex-adjusted and multivariable-adjusted Cox regressions. This association was strongest for NT-proBNP and IGFBP7 (HR 2.67, 95% CI 1.56–4.56, *P* < 0.001, and HR 1.79, 95% CI 1.32–2.43, *P* < 0.001, respectively, in the multivariable-adjusted model) (*Table 9*, *Figure 1*, Panel E).

**Table 9:** Cox regressions adjusted for log-transformed biomarkers for all-cause mortality in patients with prevalent AF

Biomarkers (*n*)^a^	HR	95% confidence interval		*P*-value
		Lower	Upper	
Adjusted for age and sex				
NT-proBNP (523)	2.67	1.56	4.56	**<0**.**001**
Angpt2 (523)	1.53	1.12	2.10	**0**.**008**
BMP10 (525)	1.44	0.97	2.13	0.072
FGF23 (525)	1.50	1.15	1.95	**0**.**002**
IGFBP7 (525)	1.79	1.32	2.43	**<0**.**001**
Multivariable adjustment^b^				
NT-proBNP (508)	2.57	1.48	4.45	**<0**.**001**
Angpt2 (508)	1.53	1.09	2.15	**0**.**014**
BMP10 (510)	1.29	0.84	1.97	0.239
FGF23 (510)	1.40	1.05	1.88	**0**.**023**
IGFBP7 (510)	1.75	1.28	2.39	**<0**.**001**

Significant regressions (*P* < 0.05) marked in bold. Analyses are based on 40 events. Combined endpoint includes: all-cause mortality, stroke/TIA, myocardial infarction, coronary heart disease, and heart failure.

Abbreviations: Angpt2, angiopoietin 2; BMP10, bone morphogenetic protein 10; FGF23, fibroblast growth factor 23; IGFBP7, insulin-like growth factor binding protein 7; NT-proBNP, N-terminal pro brain-natriuretic peptide.

^a^Numbers in parentheses are cases included in the analysis.

^b^Model adjusted for age, sex, systolic blood pressure, BMI, diabetes, dyslipidaemia, and smoking.

The predictive ability did not significantly differ between models, although a trend could be observed with a higher AUC for the biomarkers in combination with the risk factors compared to risk factors alone (AUC 0.809 and 0.760, respectively, *P*-value for difference 0.109) (*Figure 2*, Panel E, Supplementary material online, *Table S1*). NT-proBNP had the highest ROC (0.827), followed by FGF23 (0.812) and IGFBP7 (0.807) (see Supplementary material online, *Table S2* and *Figure S6*).

##### Combined cardiovascular disease outcomes in patients with AF at baseline

NT-proBNP, FGF23, and IGFB7 were significantly associated with the combined CVD outcome (all-cause mortality, stroke/TIA, myocardial infarction, coronary heart disease, heart failure) in patients with AF at baseline (*Table 10*, *Figure 1*, Panel F). This association was strongest for IGFBP7 (HR 1.48, 95% CI 1.21–1.80, *P* < 0.001), followed by NT-proBNP (HR 1.38, 95% CI 1.04–1.82, *P* = 0.024) in multivariable-adjusted Cox regressions.

**Table 10:** Cox regression for log-transformed biomarkers for the combined outcomes in patients with prevalent AF

Biomarkers (*n*)^a^	HR	95% confidence interval		*P*-value
		Lower	Upper	
Adjusted for age and sex				
NT-proBNP (517)	1.42	1.07	1.87	**0**.**014**
Angpt2 (517)	1.17	0.97	1.42	0.093
BMP10 (517)	1.17	0.93	1.47	0.176
FGF23 (517)	1.27	1.07	1.52	**0**.**008**
IGFBP7 (531)	1.49	1.23	1.81	**<0**.**001**
Multivariable adjustment^b^				
NT-proBNP (502)	1.38	1.04	1.82	**0**.**024**
Angpt2 (502)	1.14	0.95	1.14	0.173
BMP10 (504)	1.11	0.87	1.41	0.873
FGF23 (504)	1.23	1.03	1.48	**0**.**026**
IGFBP7 (504)	1.48	1.21	1.80	**<0**.**001**

Significant regressions (*P* < 0.05) marked in bold. Analyses are based on 81 events. Combined endpoint includes: all-cause mortality, stroke/TIA, myocardial infarction, coronary heart disease, and heart failure.

Abbreviations: Angpt2, angiopoietin 2; BMP10, bone morphogenetic protein 10, FGF23, fibroblast growth factor 23; IGFBP7, insulin-like growth factor binding protein 7; NT-proBNP, N-terminal pro brain-natriuretic peptide.

^a^Numbers in parentheses are cases included in the analysis.

^b^Model adjusted for age, sex, systolic blood pressure, BMI, diabetes, dyslipidaemia, and smoking.

The predictive ability did not differ significantly between the biomarker and risk factor model. The addition of biomarkers to the risk factor model improved risk prediction, with borderline statistical significance for the difference (AUC 0.691 and 0.649, respectively, 95% CI for AUC difference 0 to −0.082, *P*-value for difference 0.051) (*Figure 2*, Panel F, Supplementary material online, *Table S1*). IGFBP7 had the highest ROC (0.681), followed by FGF23 (0.678).

### Sensitivity analysis

In sensitivity analyses with additional adjustment for heart failure and AF pattern, these results remained largely robust (see Supplementary material online, *Tables S5*–*S10*). FGF23 reached statistical significance in association with incident AF but lost its significant association with all-cause mortality in individuals with incident AF, combined CVD outcomes, and all-cause mortality in individuals with AF at baseline (see Supplementary material online, *Tables S5*, *S6*, *S9*, and *S10*). IGFBP7 was no longer statistically significantly associated with incident AF and recurrent AF (see Supplementary material online, *Tables S5* and *S6*).

Subgroup analyses adjusting for kidney function are presented in Supplementary material online, *Tables S13*–*S15*). In this smaller group of patients, some associations lost statistical significance. The additional adjustment for eGFR did not markedly change associations of BMP10 or for recurrent AF (see Supplementary material online, *Table S14*).

## Discussion

We present comprehensive data on the association and predictive ability of the four emerging circulating biomarkers Angpt2, BMP10, FGF23, and IGFBP7 in comparison with the established biomarker NT-proBNP in relation to incidence and outcomes of AF in over 1000 individuals. All four novel biomarkers showed predictive value for rhythm. Angpt2, FGF23, and IGFBP7 further revealed distinct strengths in the prediction of specific outcomes in individuals with incident or prevalent AF. Higher plasma concentrations indicated an increased risk. Overall, NT-proBNP remained the strongest predictor for both rhythm and other CVD outcomes, but the predictive performance of each biomarker varied across outcomes. The predictive ability of biomarkers and risk factors was comparable in all analyses. The addition of biomarkers to clinical variables showed a trend towards improvement in model performance, although this trend only met statistical significance for all-cause mortality in incident AF.

The association of the novel biomarkers was less consistent. All four novel biomarkers were associated with rhythm across both MSA and Cox regressions. The association of the biomarkers with cardiovascular outcomes was weaker, in particular in individuals without AF at baseline. This observation highlights a potential specificity of the biomarkers for AF itself rather than for general cardiovascular risk. FGF23 was the only biomarker significantly associated with the transition to death without prior AF, indicating a more general predictive ability for mortality. Additional adjustment for heart failure and AF pattern weakened the association of FGF23 and IGFBP7, but not for any of the other biomarkers. The complexity of these results mirrors the heterogeneity of the involved pathophysiological pathways and the interdependence of risk and predictive factors.

Our findings extend previous studies, in which these four novel biomarkers have shown promising results in AF. BMP10, Angpt2, and FGF23 in combination with age, sex, and BMI helped to distinguish patients with AF from those with other cardiovascular conditions in the Birmingham and Black Country Atrial Fibrillation Registry (BBC-AF).^4^ Angpt2, BMP10, FGF23, and NT-proBNP were associated with AF during blood withdrawal, and all five biomarkers examined in this study were associated with AF burden in 508 individuals before AF ablation in the ISOLATION study.^21^ In individuals with established AF, higher plasma concentrations of BMP10 were associated with higher risk of ischaemic stroke, heart failure, and all-cause mortality in the ARISTOTLE trial^6^ and with all-cause mortality and a composite of major adverse cardiovascular events (MACE) in the Swiss-AF study.^5^ Angpt2 improved prediction of LVEF recovery after AF ablation in 208 individuals with an LVEF <50%.^22^ IGFB7 and NT-proBNP were associated with congestive heart failure hospitalizations among 3691 patients with AF.^23^ In 2987 individuals with AF, Angpt2 was independently associated with future heart failure hospitalizations and added prognostic ability in addition to clinical factors and NT-proBNP in a validation cohort with 13 079 individuals.^24^ In our analyses, adjustment for HF weakened the observed associations for FGF23 and IGFBP7. These findings suggest that HF may be a confounder or on the pathophysiological pathway towards the arrhythmia and its complications for the two biomarkers. Beyond heart failure, atrial cardiomyopathy (AtCM) has been increasingly recognized as an underlying condition of AF. As the first consensus definition of AtCM was published merely ten years ago,^25^ with a staging system first proposed in 2024,^26^ the concept still lacks sufficient clinical validation. In our analysis, Angpt2 and BMP10 were significantly associated with future AF even after multivariable adjustment. These findings suggest that these biomarkers are associated with early atrial changes and precursor states of AF independent of other comorbidities and risk factors and may thus be candidates for the characterization of AtCM.

In the EAST-AF 4 biomolecule study in AF patients with recent onset AF, a combination of 13 biomarkers (including the four novel ones and NT-proBNP) identified four cardiometabolic risk clusters.^7^ Furthermore, lower Angpt2, BMP10, and NT-proBNP were associated with an increased likelihood of sinus rhythm after 12 months of follow-up.^8^ After pulmonary vein isolation (PVI), an association with AF recurrence was observed for BMP10 in the Swiss-AF-PVI and AFACT studies^9,27^ and for Angpt2, BMP10, and NT-proBNP in both the AXAFA biomolecule study and AFAB registry.^10,28^ Furthermore, in the AXAFA study, Angpt2, BMP10, and NT-proBNP decreased after AF ablation in arrhythmia-free individuals, whereas elevated plasma concentrations 3 months post-ablation predicted recurrent AF.^29^ Our data complement these findings in an unselected cohort across the AF patient spectrum.

AF is a common and relevant comorbidity of many chronic conditions, e.g. in patients with kidney disease, heart failure, and stroke. Previous evidence suggests that these biomarkers might also guide the detection of AF as a comorbidity in disease cohorts. The correlation of biomarkers and clinical characteristics differed across the distinct settings of patients at risk, prevalent AF, and recurrent AF. Similarly, the association of biomarkers with AF varies in disease cohorts based on the type and burden of comorbidities. In individuals with mild to severe kidney disease, FGF23 was independently associated with both prevalent and incident AF.^30^ In a small case–control study, Angpt2 was significantly higher in patients with both stage 5 chronic kidney disease and AF than in either condition alone.^31^ Angpt2 and BMP10 were associated with AF in two studies among stroke patients.^32,33^ In 2085 patients from the European BIOlogy Study to TAilored Treatment in Chronic Heart Failure (BIOSTAT-CHF) cohort with chronic heart failure, BMP10 was associated with incident AF during the 9-month follow-up.^34^

The majority of studies examine disease cohorts. Evidence at population level and new onset AF is limited and inconsistent. FGF23 was associated with incident AF among 7748 individuals from the Multi-Ethnic Study of Atherosclerosis (MESA) and the Cardiovascular Health Study (CHS) after multivariable adjustments.^35^ In contrast, FGF23 was no longer associated with incident AF in 12 349 individuals free of AF at baseline in the Atherosclerosis Risk in Communities (ARIC) study after adjustment for kidney function.^36^ IGFBP7 was associated with prevalent AF and echocardiographic alterations as well as all-cause mortality in 2001 individuals from a community-based cohort.^37^ In this context, our analyses of individuals at risk of AF add a comprehensive head-to-head comparison of these four biomarkers, with a significant association for all biomarkers (apart from a non-significant association between FGF23 in multivariable-adjusted Cox regression) in both statistical approaches.

Together with the existing evidence, our data demonstrate strong associations of the four novel biomarkers Angpt2, BMP10, FGF23, and IGFBP7 with AF and AF-related complications. This aligns with previous findings, demonstrating that a biomarker panel (composed of different biomarkers) improved risk prediction for adverse outcomes in AF.^38^ Such biomarker panels hold a promising potential for future clinical applications. An ongoing randomized controlled trial (NCT03753490) is currently evaluating biomarker-based decision support for stroke prevention with oral anticoagulations.^39^ Although biomarker panels may hold the potential for clinical applications, there is no strong evidence to support the use of biomarker-guided AF management using these distinct markers in clinical practice as of now. Until a clinical benefit of the application of biomarkers for decision making is proven, clinicians must rely on established risk stratification tools. The 2024 ESC guidelines currently recommend the use of only two biomarkers, NT-proBNP and troponin, for stroke and bleeding risk stratification in selected patients.^40,41^ A deeper understanding of the pathways and mechanisms in which these biomarkers are involved in AtCM and AF may provide pathophysiological insights and possibly future therapeutic targets.

## Limitations and strengths

The cohort analysed in this study is a single-centre cohort, and external validation of our findings is required. Whereas AF burden is increasingly recognized as an important marker of disease severity and predictor of AF outcomes in recent years,^42–44^ this type of information is not available in the clinical setting reflected by our cohort due to a lack of continuous monitoring. We therefore rely on the established AF classification, and cannot explore the association of this emerging biological marker with circulating biomarkers in this analysis. Some subgroup analyses, in particular outcomes in patients with incident AF, are limited by the relatively small number of events, which may have reduced the power to detect associations and differences. In the predictive models, a consistent trend could be observed, that the addition of the biomarkers to the risk factor model improved model performance, although without reaching statistical significance in most analyses. Due to the size of the subgroups, we limited the number of covariates for multivariable adjustment. Residual confounding thus needs to be considered. Our analyses have not been adjusted for multiple testing and are thus explorative. Nevertheless, a major strength of this analysis is the relatively large sample size in this well-phenotyped prospective clinical cohort across a broad population of patients at risk of AF or with manifest disease that reflects the heterogeneity of AF in clinical practice.

## Conclusions

The novel biomarkers Angpt2, BMP10, FGF 23, and IGFBP7 showed varying predictive ability across the AF disease spectrum from disease onset over recurrence and adverse cardiovascular outcomes and mortality. All biomarkers were associated with AF incidence and recurrence. Thus, their potential for clinical application should be investigated further.

## Supplementary Material

euag096_Supplementary_Data

## Data Availability

The data underlying this article will be shared on reasonable request to the corresponding author.
