# Supplementary material for: Comprehensive assessment of novel cardiovascular biomarkers in AF
Source: Europace. 2026 May 15;28(5):euag096. doi: 10.1093/europace/euag096 (PMC13199998; doi:10.1093/europace/euag096)
Supplement: euag096_Supplementary_Data [file euag096_supplementary_data.docx]

**Supplement**

Comprehensive assessment of novel cardiovascular biomarkers in AF – Ohlrogge *et al.*

| Supplement figure 1: Correlation matrix of biomarkers with each other and baseline parameters | p. 1 |
| --- | --- |
| Supplement figures 2-7: Performance metrics of the multivariate prediction models for each biomarker | **p. 2** |
| Supplement table 1: Area under the ROC (AUC) curve for (I) the combined biomarkers, (II) risk factors and (III) a combined model of biomarkers and risk factors | **p. 8** |
| Supplement table 2: Performance metrics of the multivariate prediction models for each biomarker | **p. 10** |
| Supplement table 3 and 4: Outcomes in recurrent AF | **p. 12** |
| Supplement table 5 – 10: Sensitivity analysis for heart failure | **p. 13** |
| Supplement table 11 – 16: Sensitivity analysis for kidney function | **p. 15** |


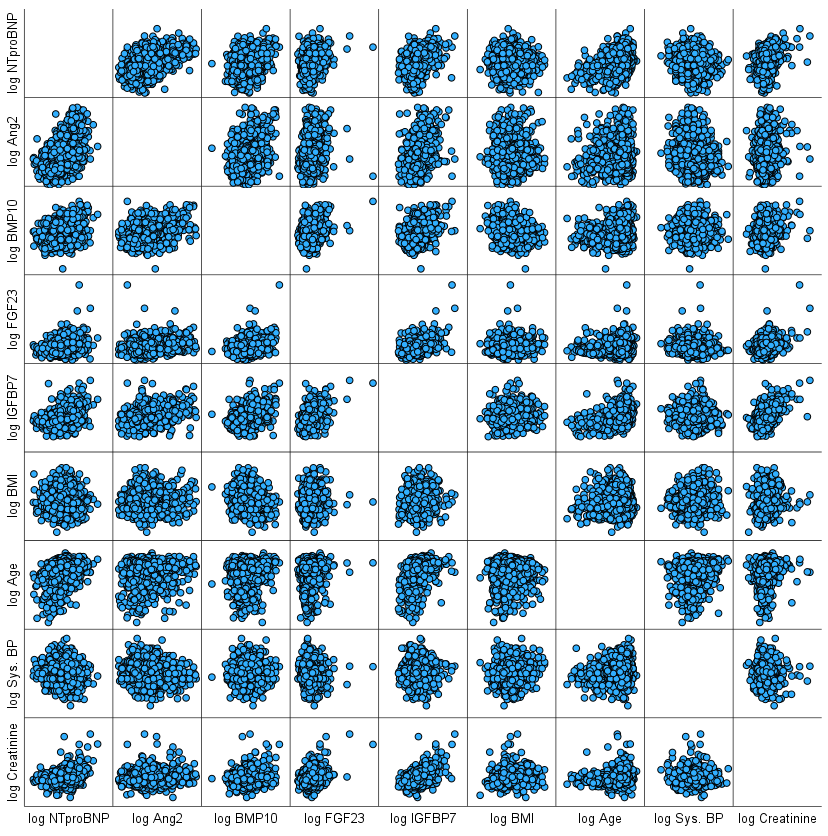


Supplement figure 1 – Correlation matrix of biomarkers with each other and baseline parameters. Abbreviations: Ang2 – angiopoietin 2, BMI – body mass index, BMP – bone morphogenetic protein 10, BP – blood pressure, FGF23 – fibroblast growth factor 23, IGFBP7 - insulin-like growth factor binding protein 7, NTproBNP - N-terminal pro B-type natriuretic peptide


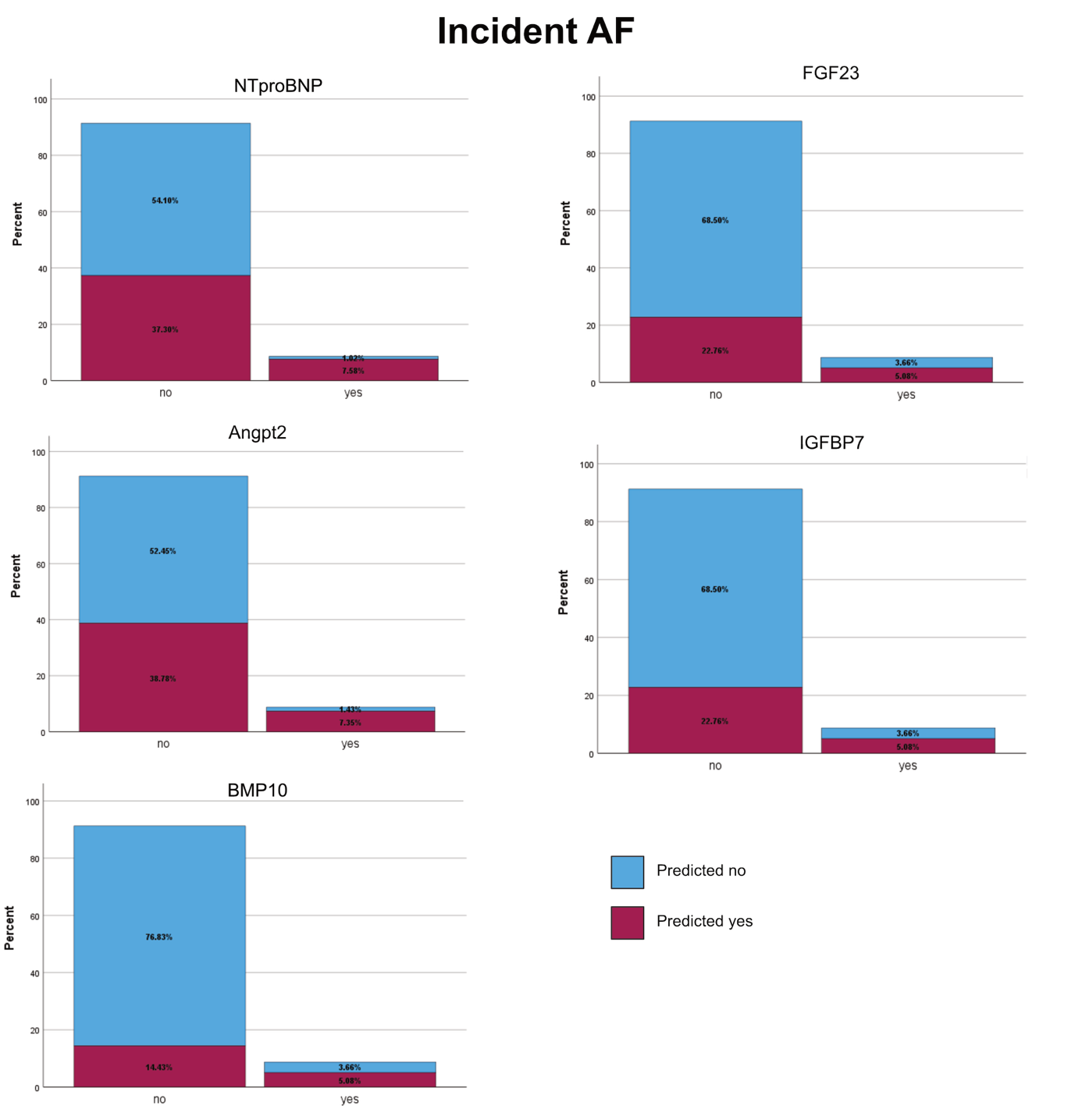


Supplement figure 2 – Performance metrics of the multivariate prediction models for each biomarker for incident AF. Abbreviations: Ang2 – angiopoietin 2, BMI – body mass index, BMP – bone morphogenetic protein 10, BP – blood pressure, FGF23 – fibroblast growth factor 23, IGFBP7 - insulin-like growth factor binding protein 7, NTproBNP - N-terminal pro B-type natriuretic peptide


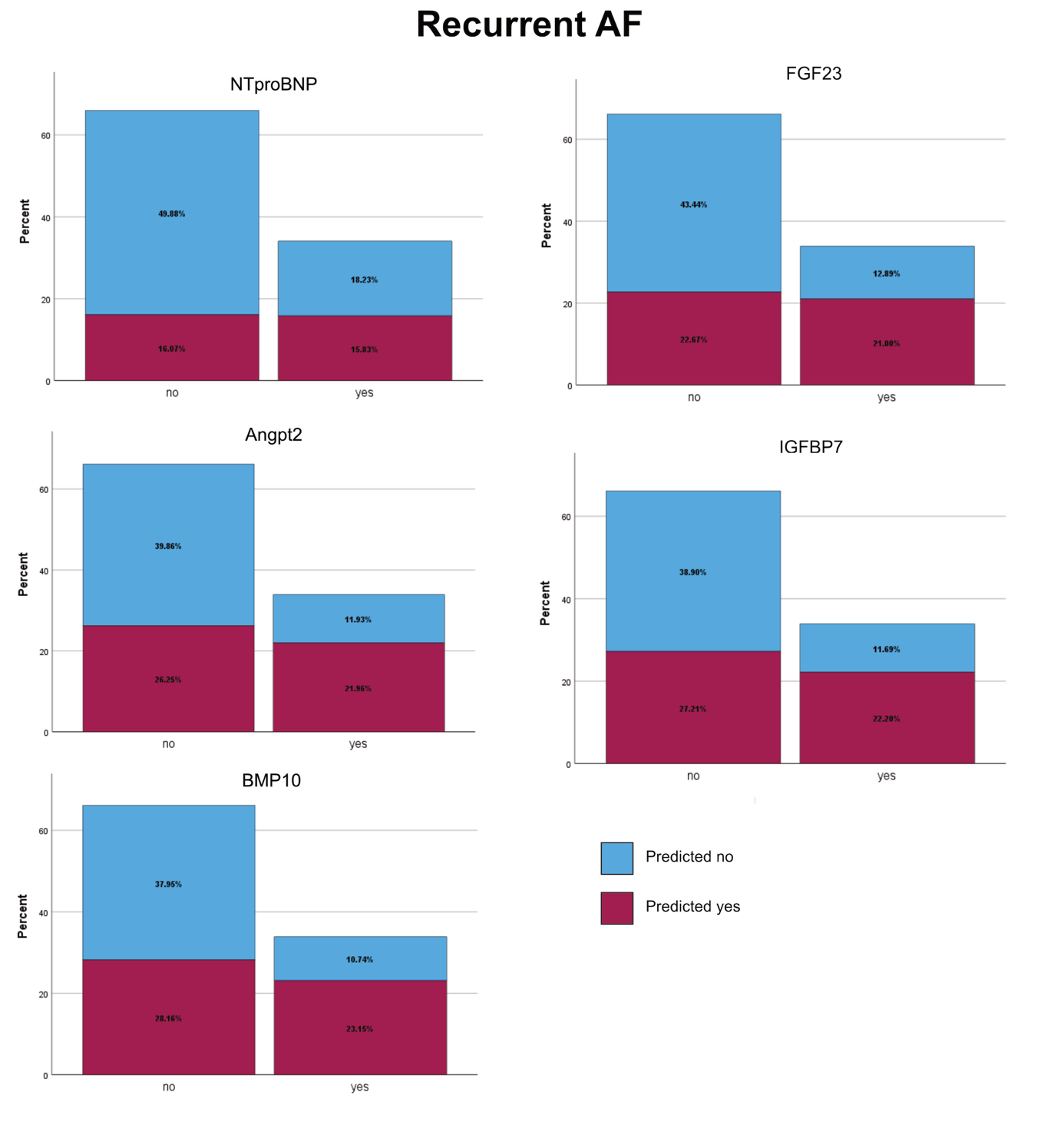


Supplement figure 3 – Performance metrics of the multivariate prediction models for each biomarker for incident AF. Abbreviations: Ang2 – angiopoietin 2, BMI – body mass index, BMP – bone morphogenetic protein 10, BP – blood pressure, FGF23 – fibroblast growth factor 23, IGFBP7 - insulin-like growth factor binding protein 7, NTproBNP - N-terminal pro B-type natriuretic peptide


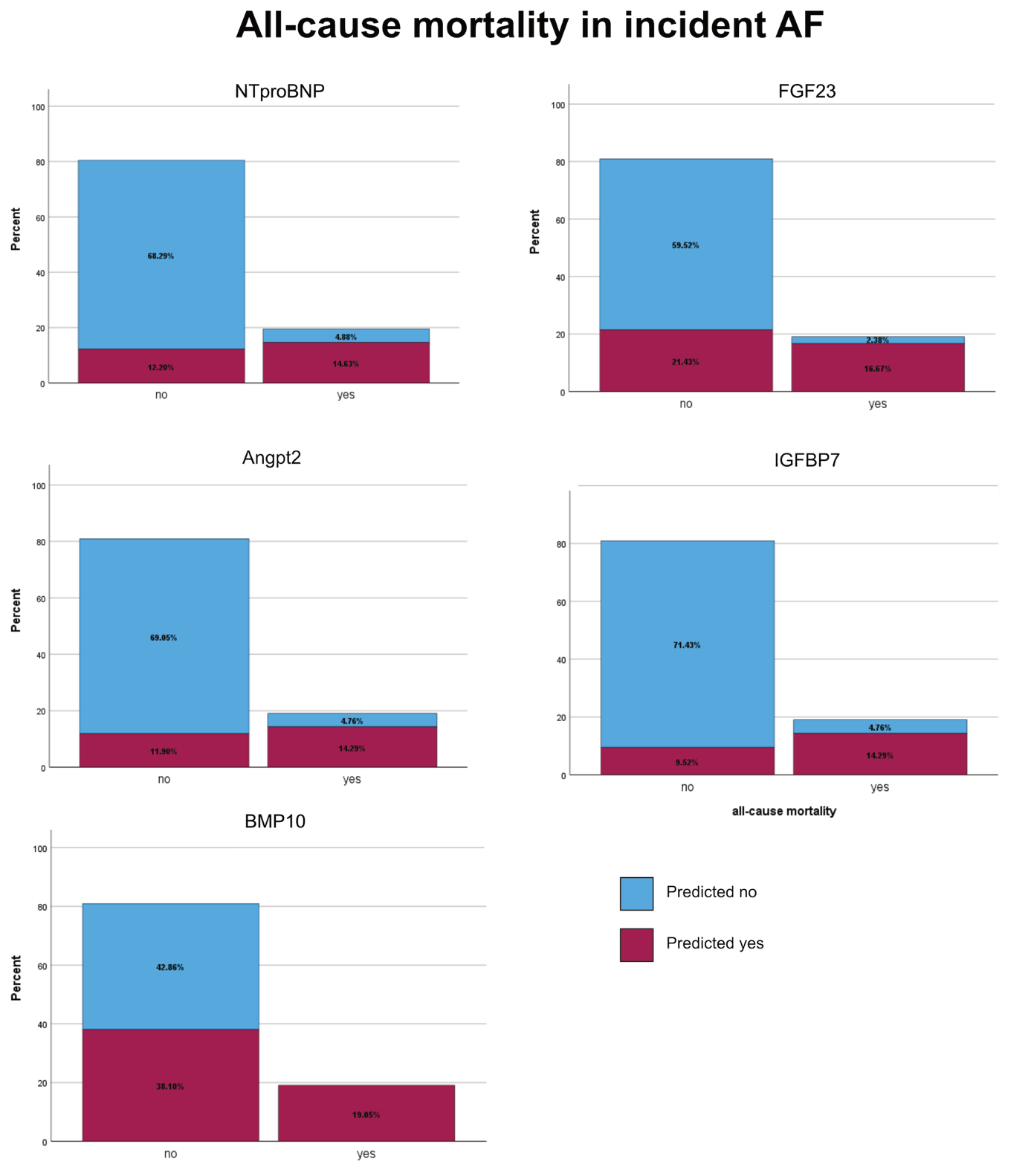


Supplement figure 4 – Performance metrics of the multivariate prediction models for each biomarker for incident AF. Abbreviations: Ang2 – angiopoietin 2, BMI – body mass index, BMP – bone morphogenetic protein 10, BP – blood pressure, FGF23 – fibroblast growth factor 23, IGFBP7 - insulin-like growth factor binding protein 7, NTproBNP - N-terminal pro B-type natriuretic peptide


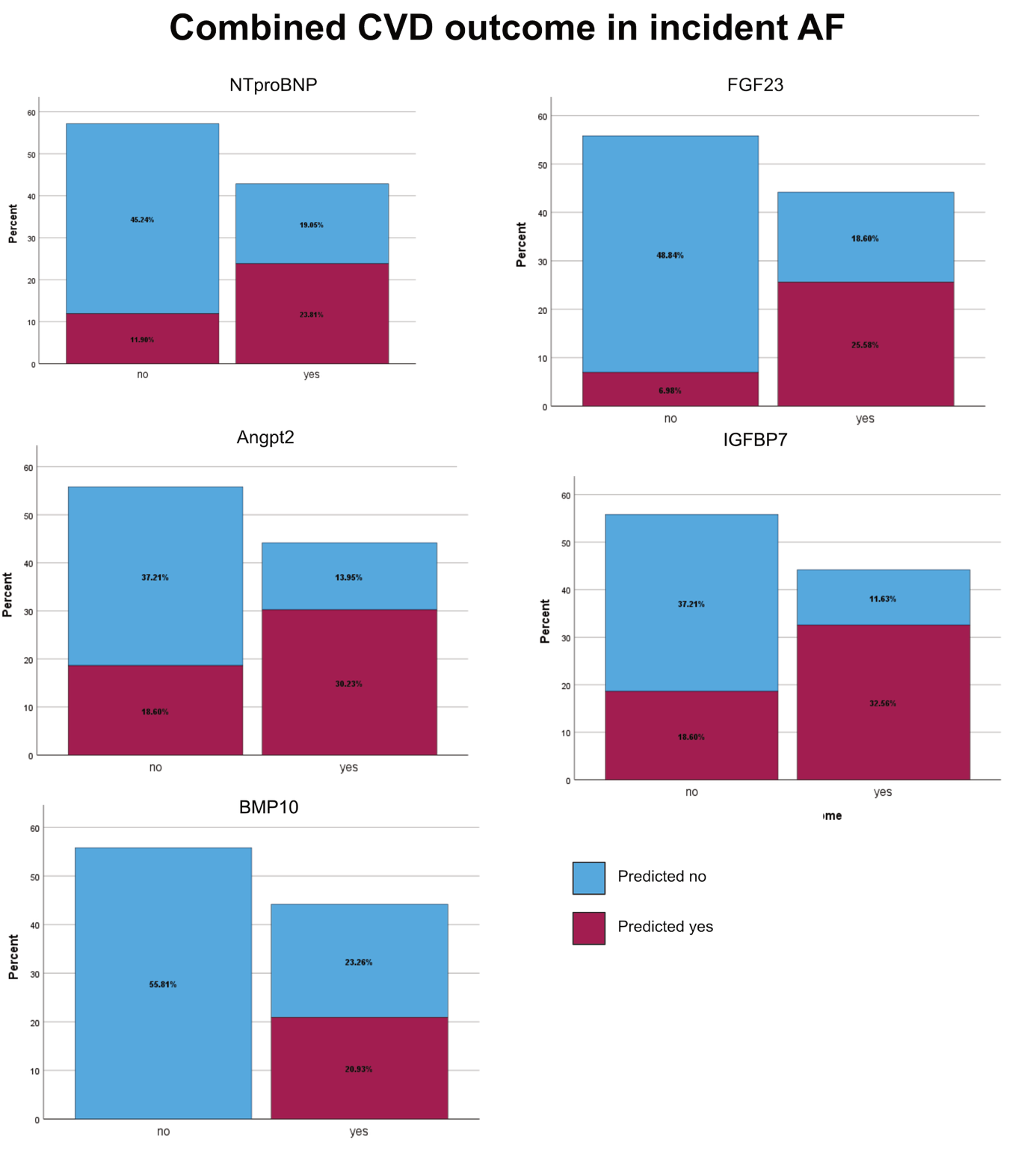


Supplement figure 5 – Performance metrics of the multivariate prediction models for each biomarker for incident AF. Abbreviations: Ang2 – angiopoietin 2, BMI – body mass index, BMP – bone morphogenetic protein 10, BP – blood pressure, FGF23 – fibroblast growth factor 23, IGFBP7 - insulin-like growth factor binding protein 7, NTproBNP - N-terminal pro B-type natriuretic peptide


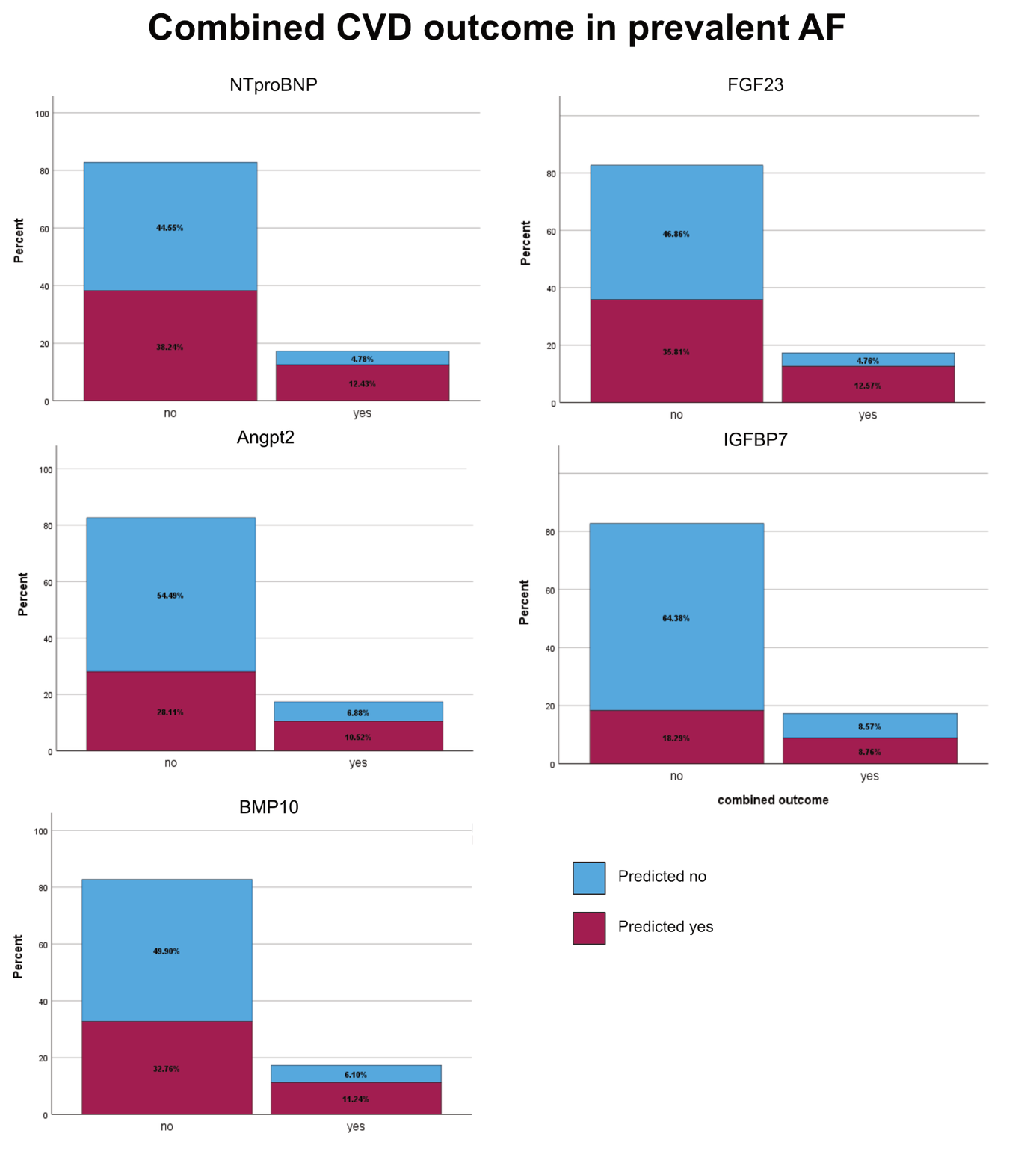


Supplement figure 6 – Performance metrics of the multivariate prediction models for each biomarker for incident AF. Abbreviations: Ang2 – angiopoietin 2, BMI – body mass index, BMP – bone morphogenetic protein 10, BP – blood pressure, FGF23 – fibroblast growth factor 23, IGFBP7 - insulin-like growth factor binding protein 7, NTproBNP - N-terminal pro B-type natriuretic peptide


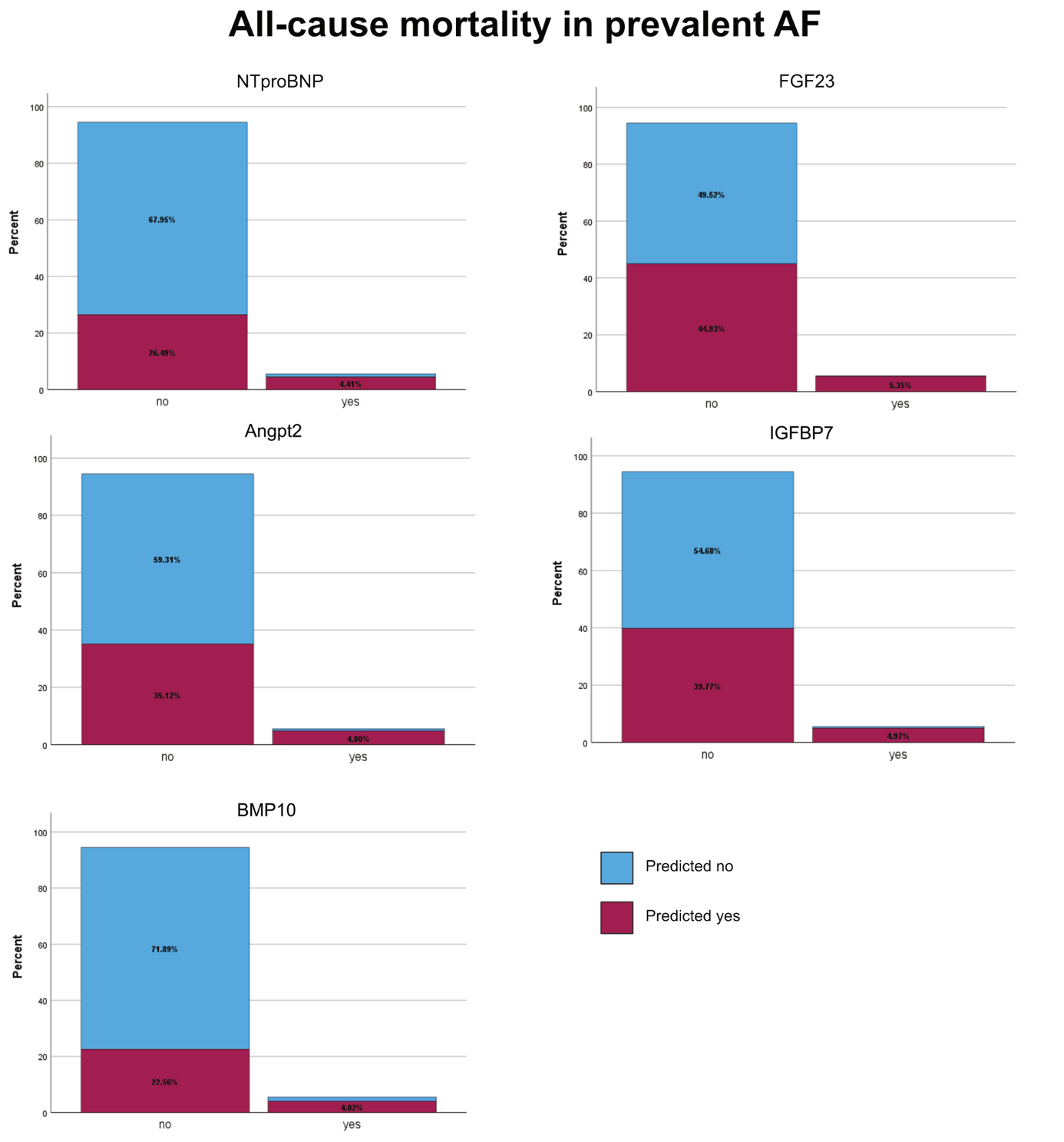


Supplement figure 7 – Performance metrics of the multivariate prediction models for each biomarker for incident AF. Abbreviations: Ang2 – angiopoietin 2, BMI – body mass index, BMP – bone morphogenetic protein 10, BP – blood pressure, FGF23 – fibroblast growth factor 23, IGFBP7 - insulin-like growth factor binding protein 7, NTproBNP - N-terminal pro B-type natriuretic peptide

|  | AUC | AUC Differences | 95% confidence interval of AUC differences | | p-value |
| --- | --- | --- | --- | --- | --- |
|  | | | lower | upper |  |
| Incident AF | | | | | |
| Risk factors | 0.606 | - | - | - | - |
| CHA₂DS₂-VA Score | 0.604 |  |  |  |  |
| Biomarkers combined | 0.643 | - | - | - | - |
| Combined model | 0.652 | - | - | - | - |
| Combined biomarkers – risk factors | - | 0.037 | -0.041 | 0.115 | 0.355 |
| Combined biomarkers – combined model | - | -0.009 | -0.044 | 0.026 | 0.618 |
| Risk factors – combined model | - | -0.046 | -0.100 | 0.008 | 0.098 |
| All-cause mortality in incident AF | | | | | |
| Risk factors | 0.766 | - | - | - | - |
| CHA₂DS₂-VA Score | 0.644 |  |  |  |  |
| Combined biomarker | 0.873 | - | - | - | - |
| Combined model | 0.904 | - | - | - | - |
| Combined biomarkers – risk factors | - | 0.107 | -0.030 | 0.244 | 0.127 |
| Combined biomarkers – combined model | - | -0.031 | -0.065 | 0.003 | 0.077 |
| Risk factors – combined model | - | -0.137 | -0.268 | -0.007 | 0.039 |
| Combined CVD outcome in incident AF | | | | | |
| Risk factors | 0.690 | - | - | - | - |
| CHA₂DS₂-VA Score | 0.513 |  |  |  |  |
| Combined biomarker | 0.684 | - | - | - | - |
| Combined model | 0.744 | - | - | - | - |
| Combined biomarkers – risk factors | - | -0.006 | -0.178 | 0.166 | 0.944 |
| Combined biomarkers – combined model | - | -0.060 | -0.172 | 0.051 | 0.288 |
| Risk factors – combined model | - | -0.054 | -0.149 | 0.041 | 0.262 |
| Recurrent AF | | | | | |
| Risk factors | 0.625 | - | - | - | - |
| CHA₂DS₂-VA Score | 0.534 |  |  |  |  |
| Combined biomarker | 0.616 | - | - | - | - |
| Combined model | 0.666 | - | - | - | - |
| Combined biomarkers – risk factors | - | .008 | -.066 | .083 | .825 |
| Combined biomarkers – combined model | - | -.041 | -.087 | .005 | .079 |
| Risk factors – combined model | - | -.050 | -.094 | -.005 | .031 |
| All-cause mortality in prevalent AF | | | | | |
| Risk factors | 0.760 | - | - | - | - |
| CHA₂DS₂-VA Score | 0.718 |  |  |  |  |
| Combined biomarker | 0.781 | - | - | - | - |
| Combined model | 0.809 | - | - | - | - |
| Combined biomarkers – risk factors | - | 0.031 | -0.061 | 0.122 | 0.505 |
| Combined biomarkers – combined model | - | -0.023 | -0.066 | 0.009 | 0.155 |
| Risk factors – combined model | - | -0.050 | -0.121 | 0.013 | 0.109 |
| Combined CVD outcome in prevalent AF | | | | | |
| Risk factors | 0.649 | - | - | - | - |
| Combined biomarker | 0.673 | - | - | - | - |
| CHA₂DS₂-VA Score | 0.613 |  |  |  |  |
| Combined model | 0.691 | - | - | - | - |
| Combined biomarkers – risk factors | - | 0.019 | -0.031 | 0.072 | 0.450 |
| Combined biomarkers – combined model | - | -0.014 | -0.046 | 0.006 | 0.179 |
| Risk factors – combined model | - | -0.046 | -0.082 | 0.000 | 0.051 |

Supplement table 1 - Area under the ROC (AUC) curve and paired-samples area differences under the ROC curves for (I) the CHA₂DS₂-VA Score, (II) the combined biomarkers (NTproBNP, Ang2, BMP10, FGF23, IGFBP7), (III) risk factors (age, sex, systolic blood pressure, BMI, diabetes, dyslipidaemia and smoking) and (I) a combined model of biomarkers and risk factors.

Abbreviations: Angpt2 – angiopoietin 2, BMP10 – bone morphogenetic protein 10, CHA_2_DS_2_-VA-Score – congestive heart failure (1), hypertension (1), age ≥75 years (2), diabetes mellitus (1), stroke/TIA (1), vascular disease (1), age 65-74 years (1); FGF23 – fibroblast growth factor 23, IGFBP7 - insulin-like growth factor binding protein 7, NTproBNP - N-terminal pro brain-natriuretic peptide, ROC – Receiver operating characteristic.

| Biomarker | ROC | Youden’s index | Cut off | Adjusted R^2^ |
| --- | --- | --- | --- | --- |
| Incident AF | | | | |
| NTproBNP | 0.751 | 0.473 | 0.06799 | 0.137 |
| Angpt2 | 0.731 | 0.412 | 0.07385 | 0.109 |
| BMP10 | 0.724 | 0.423 | 0.131012 | 0.109 |
| FGF23 | 0.696 | 0.392 | 0.089068 | 0.086 |
| IGFBP7 | 0.697 | 0.332 | 0.1075684 | 0.089 |
| All-cause mortality in incident AF | | | | |
| NTproBNP | 0.856 | 0.598 | 0.2510936 | 0.415 |
| Angpt2 | 0.794 | 0.603 | 0.2577409 | 0.300 |
| BMP10 | 0.768 | 0.529 | 0.1026692 | 0.225 |
| FGF23 | 0.816 | 0.610 | 0.2102914 | 0.274 |
| IGFBP7 | 0.838 | 0.632 | 0.2526161 | 0.322 |
| Combined CVD outcome in incident AF | | | | |
| NTproBNP | 0.674 | 0.347 | 0.4959089 | 0.107 |
| Angpt2 | 0.686 | 0.351 | 0.4193964 | 0.140 |
| BMP10 | 0.697 | 0.474 | 0.6194074 | 0.131 |
| FGF23 | 0.721 | 0.454 | 0.5764251 | 0.155 |
| IGFBP7 | 0.697 | 0.404 | 0.4298003 | 0.179 |
| Recurrent AF | | | | |
| NTproBNP | 0.633 | 0.214 | 0.34967 | 0.075 |
| Angpt2 | 0.621 | 0.250 | 0.3303484 | 0.067 |
| BMP10 | 0.645 | 0.259 | 0.3295882 | 0.069 |
| FGF23 | 0.662 | 0.280 | 0.358759 | 0.073 |
| IGFBP7 | 0.632 | 0.240 | 0.321057 | 0.056 |
| All-cause mortality in prevalent AF | | | | |
| NTproBNP | 0.827 | 0.516 | 0.0514783 | 0.226 |
| Angpt2 | 0.792 | 0.416 | 0.0443299 | 0.191 |
| BMP10 | 0.786 | 0.488 | 0.0739472 | 0.174 |
| FGF23 | 0.812 | 0.499 | 0.0400135 | 0.199 |
| IGFBP7 | 0.807 | 0.476 | 0.0370014 | 0.203 |
| Combined CVD outcome in prevalent AF | | | | |
| NTproBNP | 0.656 | 0.266 | 0.1601205 | 0.081 |
| Angpt2 | 0.660 | 0.267 | 0.194759 | 0.084 |
| BMP10 | 0.651 | 0.261 | 0.1806428 | 0.074 |
| FGF23 | 0.678 | 0.301 | 0.1635729 | 0.093 |
| IGFBP7 | 0.681 | 0.289 | 0.2103681 | 0.106 |

Supplement table 2 - Performance metrics of the multivariate prediction models for each biomarker. Abbreviations: Angpt2 – angiopoietin 2, BMP10 – bone morphogenetic protein 10, FGF23 – fibroblast growth factor 23, IGFBP7 - insulin-like growth factor binding protein 7, NTproBNP - N-terminal pro brain-natriuretic peptide, ROC – Receiver operating characteristic.

**Outcomes in recurrent AF**

| Biomarkers (N)* | HR | 95% Confidence interval | | p-value |
| --- | --- | --- | --- | --- |
|  |  | **lower** | **upper** |  |
| Adjusted for age and sex | | | | |
| NTproBNP (N=134) | 1.47 | 0.78 | 2.74 | 0.232 |
| Ang2 (N=134) | 1.55 | 0.86 | 2.77 | 0.142 |
| BMP10 (N=134) | 1.07 | 0.60 | 1.92 | 0.814 |
| FGF 23 (N=133) | 1.21 | 0.90 | 1.91 | 0.415 |
| IGFBP7 (N=134) | 1.57 | 0.87 | 2.86 | 0.137 |
| Multivariable adjustment** | | | | |
| NTproBNP (N=133) | 1.56 | 0.79 | 3.08 | 0.197 |
| Ang2 (N=133) | 1.57 | 0.87 | 2.84 | 0.137 |
| BMP10 (N=133) | 1.13 | 0.60 | 2.13 | 0704 |
| FGF 23 (N=133) | 1.23 | 0.76 | 1.98 | 0.395 |
| IGFBP7 (N=135) | 1.70 | 0.91 | 3.20 | 0.099 |

**Supplement table 3 -** Cox regressions for log-transformed biomarkers in relation to combined outcomes in patients with recurrent AF. Note: Statistically significant regressions (p <0.05) marked in bold. Analyses are based on 14 events. *Numbers in parentheses are cases included in the analysis. **Model adjusted for age, sex, systolic blood pressure, BMI, diabetes, dyslipidaemia and smoking. Combined endpoints include: all-cause mortality, stroke/TIA, myocardial infarction, coronary heart disease, heart failure. Abbreviations: Angpt2 – angiopoietin 2, BMP10 – bone morphogenetic protein 10, FGF23 – fibroblast growth factor 23, IGFBP7 - insulin-like growth factor binding protein 7, NTproBNP - N-terminal pro brain-natriuretic peptide

| Biomarkers (N)* | HR | 95% Confidence interval | | p-value |
| --- | --- | --- | --- | --- |
|  |  | **lower** | **upper** |  |
| Adjusted for age and sex | | | | |
| NTproBNP (N=140) | 4.31 | 1.48 | 12.58 | **0.008** |
| Ang2 (N=140) | 2.73 | 1.62 | 6.56 | **0.025** |
| BMP10 (N=140) | 1.55 | 0.69 | 3.45 | 0.286 |
| FGF 23 (N=139) | 0.98. | 0.42 | 2.26 | 0.957 |
| IGFBP7 (N=140) | 2.10 | 0.82 | 5.42 | 0.123 |
| Multivariable adjustment** | | | | |
| NTproBNP (N=139) | 3.11 | 1.11 | 8.74 | **0.032** |
| Ang2 (N=139) | 3.16 | 1.00 | 9.97 | **0.049** |
| BMP10 (N=139) | 1.26 | 0.49 | 3.23 | 0.635 |
| FGF 23 (N=138) | 0.83 | 0.03 | 2.19 | 0.698 |
| IGFBP7 (N=143) | 2.69 | 0.80 | 9.04 | 0.109 |

**Supplement table 4 -** Cox regressions for log-transformed biomarkers in relation to all-cause mortality in patients with recurrent AF. Note: Statistically significant regressions (p <0.05) marked in bold. Analyses are based on 7 events. *Numbers in parentheses are cases included in the analysis. **Model adjusted for age, sex, systolic blood pressure, BMI, diabetes, dyslipidaemia and smoking. Combined endpoints include: all-cause mortality, stroke/TIA, myocardial infarction, coronary heart disease, heart failure. Abbreviations: Angpt2 – angiopetin 2, BMP10 – bone morphogenetic protein 10, FGF23 – fibroblast growth factor 23, IGFBP7 - insulin-like growth factor binding protein 7, NTproBNP - N-terminal pro brain-natriuretic peptide

**Sensitivity analyses**

**Heart failure**

**For incident AF:**

| Biomarkers (N)* | HR | 95% Confidence interval | | p-value |
| --- | --- | --- | --- | --- |
|  |  | lower | upper |  |
| Multivariable adjustment** | | | | |
| NTproBNP (N=486) | 2.10 | 1.39 | 3.17 | **<0.001** |
| Angpt2 (N=488) | 1.58 | 1.14 | 2.17 | **0.006** |
| BMP10 (N=490) | 1.56 | 1.10 | 2.23 | **0.013** |
| FGF23 (N=490) | 1.36 | 1.02 | 1.72 | **0.036** |
| IGFBP7 (N=490) | 1.38 | 0.98 | 1.95 | 0.069 |

**Supplement table 5** - Cox regressions for log-transformed biomarker concentrations in relation to incident AF in the at risk cohort. Statistically significant regressions (p<0.05) are marked in bold. Analyses are based on 42 events. *Numbers in parentheses are cases included in the analysis. ** Multivariable-adjusted model: age, sex, systolic blood pressure, BMI, diabetes, dyslipidaemia, smoking, heart failure. Abbreviations: Angpt2 – angiopoietin 2, BMP10 – bone morphogenetic protein 10, FGF23 – fibroblast growth factor 23, IGFBP7 - insulin-like growth factor binding protein 7, NTproBNP - N-terminal pro brain-natriuretic peptide

**For all-cause mortality in patients with incident AF:**

| Biomarkers (N)* | HR | 95% Confidence interval | | p-value |
| --- | --- | --- | --- | --- |
|  |  | **lower** | **upper** |  |
| Multivariable adjustment** | | | | |
| NTproBNP (N=38) | 4.50 | 1.28 | 15.8 | **0.019** |
| Angpt2 (N=39) | 6.74 | 1.02 | 45.0 | **0.049** |
| BMP10 (N=39) | 1.89 | 0.56 | 6.38 | 0.305 |
| FGF23 (N=39) | 3.23 | 0.99 | 10.54 | 0.052 |
| IGFBP7 (N=39) | 2.77 | 1.12 | 6.42 | **0.031** |

**Supplement table 6 -** Cox regressions for log-transformed biomarkers in relation to all-cause mortality in patients with incident AF. Note. Significant regressions (p <0.05) marked in bold. Analyses are based on 8 events. *Numbers in parentheses are cases included in the analysis. **Model adjusted for age, sex, systolic blood pressure, BMI, diabetes, dyslipidaemia, smoking and heart failure. Abbreviations: Angpt2 – angiopoietin 2, BMP10 – bone morphogenetic protein 10, FGF23 – fibroblast growth factor 23, IGFBP7 - insulin-like growth factor binding protein 7, NTproBNP - N-terminal pro brain-natriuretic peptide

**For combined CVD outcomes in patients with incident AF:**

| Biomarkers (N)* | HR | 95% Confidence interval | | p-value |
| --- | --- | --- | --- | --- |
| Multivariable adjustment** | | | | |
| NTproBNP (N=41) | 2.35 | 1.08 | 5.12 | **0.032** |
| Angpt2 (N=42) | 1.81 | 0.93 | 3.53 | 0.079 |
| BMP10 (N=42) | 0.82 | 0.42 | 1.60 | 0.552 |
| FGF23 (N=42) | 2.34 | 0.88 | 6.19 | 0.088 |
| IGFBP7 (N=42) | 1.05 | 0.59 | 1.85 | 0.874 |

**Supplement table 7 -** Cox regression of log-transformed biomarkers in relation to combined outcomes in patients with incident AF. Statistically significant regression models (p<0.05) are marked in bold. Analyses are based on 19 events. *Numbers in parentheses are cases included in the analysis. **Model adjusted for age, sex, systolic blood pressure, BMI, diabetes, dyslipidaemia, smoking and heart failure. Combined endpoint includes: all-cause mortality, stroke/TIA, myocardial infarction, coronary heart disease, heart failure. Abbreviations: Angpt2 – angiopoietin 2, BMP10 – bone morphogenetic protein 10, FGF23 – fibroblast growth factor 23, IGFBP7 - insulin-like growth factor binding protein 7, NTproBNP - N-terminal pro brain-natriuretic peptide

**For recurrent AF:**

| Biomarkers (N)* | HR | 95% Confidence interval | | p-value |
| --- | --- | --- | --- | --- |
|  |  | **lower** | **upper** |  |
| Multivariable adjustment** | | | | |
| NTproBNP (N=396) | 1.27 | 1.00 | 1.62 | **0.050** |
| Angpt2 (N=397) | 1.19 | 0.99 | 1.38 | **0.063** |
| BMP10 (N=397) | 1.19 | 0.96 | 1.36 | **0.112** |
| FGF23 (N=393) | 1.23 | 1.01 | 1.49 | **0.041** |
| IGFBP7 (N=396) | 1.17 | 0.93 | 1.48 | 0.171 |

**Supplement table 8** -Cox regressions for log-transformed biomarkers for recurrent AF in the high risk cohort. Note. Significant regressions (p <0.05) marked in bold. Analyses are based on 138 events. *Numbers in parentheses are cases included in the analysis. **Model adjusted for age, sex, systolic blood pressure, BMI, diabetes, dyslipidaemia, smoking and heart failure. Abbreviations: Angpt2 – angiopoietin 2, BMP10 – bone morphogenetic protein 10, FGF23 – fibroblast growth factor 23, IGFBP7 - insulin-like growth factor binding protein 7, NTproBNP - N-terminal pro brain-natriuretic peptide

**For combined CVD outcomes in patients with AF at baseline:**

| Biomarkers (N)* | HR | 95% Confidence interval | | p-value |
| --- | --- | --- | --- | --- |
|  |  | **lower** | **upper** |  |
| Multivariable adjustment** | | | | |
| NTproBNP (N=495) | 1.44 | 1.05 | 1.98 | 0.025 |
| Angpt2 (N=495) | 1.17 | 0.95 | 1.44 | 0.139 |
| BMP10 (N=497) | 1.04 | 0.80 | 1.35 | 0.753 |
| FGF23 (N=497) | 1.20 | 0.98 | 1.47 | 0.077 |
| IGFBP7 (N=497) | 1.51 | 1.23 | 1.86 | **<0.001** |

**Supplement table 9 -** Cox regression for log-transformed biomarkers for the combined outcomes in patients with prevalent AF. Note. Significant regressions (p < 0.05) marked in bold. Analyses are based on 80 events *Numbers in parentheses are cases included in the analysis. **Model adjusted for age, sex, systolic blood pressure, BMI, diabetes, dyslipidaemia, smoking, AF type and heart failure. Combined endpoint includes: all-cause mortality, stroke/TIA, myocardial infarction, coronary heart disease, heart failure. Abbreviations: Angpt2 – angiopoietin 2, BMP10 – bone morphogenetic protein 10, – fibroblast growth factor 23, IGFBP7 - insulin-like growth factor binding protein 7, NTproBNP - N-terminal pro brain-natriuretic peptide

**For all-cause mortality in patients with AF at baseline:**

| Biomarkers (N)* | HR | 95% Confidence interval | | p-value |
| --- | --- | --- | --- | --- |
|  |  | **lower** | **upper** |  |
| Multivariable adjustment** | | | | |
| NTproBNP (N=501) | 2.73 | 1.46 | 5.10 | **0.002** |
| Angpt2 (N=502) | 1.61 | 1.124 | 2.33 | **0.011** |
| BMP10 (N=503) | 1.18 | 0.75 | 1.86 | 0.487 |
| FGF23 (N=503) | 1.33 | 0.95 | 1.85 | 0.097 |
| IGFBP7 (N=5) | 1.74 | 1.81 | 2.51 | **<0.001** |

**Supplement table 10** - Cox regressions adjusted for log-transformed biomarkers for all-cause mortality in patients with prevalent AF. Note. Significant regressions (p <0.05) marked in bold. Analyses are based on 26 events. *Numbers in parentheses are cases included in the analysis. **Model adjusted for age, sex, systolic blood pressure, BMI, diabetes, dyslipidaemia, smoking, AF type and heart failure. Abbreviations: Angpt2 – angiopoietin 2, BMP10 – bone morphogenetic protein 10, FGF23 – fibroblast growth factor 23, IGFBP7 - insulin-like growth factor binding protein 7, NTproBNP - N-terminal pro brain-natriuretic peptide

**Kidney function**

**For incident AF:**

| Biomarkers (N)* | HR | 95% Confidence interval | | p-value |
| --- | --- | --- | --- | --- |
|  |  | lower | upper |  |
| Multivariable adjustment** | | | | |
| NTproBNP (486) | 2.54 | 1.65 | 3.89 | **<0.001** |
| Angpt2 (488) | 1.69 | 1.23 | 2.34 | **0.001** |
| BMP10 (490) | 1.65 | 1.13 | 2.40 | **0.009** |
| FGF23 (490) | 1.27 | 0.99 | 1.65 | 0.065 |
| IGFBP7 (490) | 1.46 | 0.99 | 2.14 | 0.053 |

**Supplement table 11** - Cox regressions for log-transformed biomarker concentrations in relation to incident AF in the at risk cohort. Statistically significant regressions (p<0.05) are marked in bold. Analyses are based on 42 events. *Numbers in parentheses are cases included in the analysis. ** Multivariable-adjusted model: age, sex, systolic blood pressure, BMI, diabetes, dyslipidaemia, smoking and eGFR. Abbreviations: Angpt2 – angiopoietin 2, BMP10 – bone morphogenetic protein 10, FGF23 – fibroblast growth factor 23, IGFBP7 - insulin-like growth factor binding protein 7, NTproBNP - N-terminal pro brain-natriuretic peptide

**For all-cause mortality in patients with incident AF:**

| Biomarkers (N)* | HR | 95% Confidence interval | | p-value |
| --- | --- | --- | --- | --- |
|  |  | **lower** | **upper** |  |
| Adjusted for age, sex & eGFR | | | | |
| NTproBNP (39) | 4.77 | 1.26 | 18.06 | **0.021** |
| Angpt2 (39) | 2.37 | 1.04 | 5.38 | **0.040** |
| BMP10 (39) | 1.23 | 0.49 | 3.10 | 0.660 |
| FGF 23 (39) | 2.71 | 0.94 | 7.84 | 0.065 |
| IGFBP7 (39) | 2.12 | 1.00 | 4.50 | 0.051 |

**Supplement table 12 -** Cox regressions for log-transformed biomarkers in relation to all-cause mortality in patients with incident AF. Note. Significant regressions (p <0.05) marked in bold. Analyses are based on 8 events. *Numbers in parentheses are cases included in the analysis. Due to the low event rate no multivariable adjustment was performed, by adjustment for age, sex and eGFR. Abbreviations: Angpt2 – angiopoietin 2, BMP10 – bone morphogenetic protein 10, FGF23 – fibroblast growth factor 23, IGFBP7 - insulin-like growth factor binding protein 7, NTproBNP - N-terminal pro brain-natriuretic peptide

**For combined CVD outcomes in patients with incident AF:**

| Biomarkers (N)* | HR | 95% Confidence interval | | p-value |
| --- | --- | --- | --- | --- |
| Multivariable adjustment** | | | | |
| NTproBNP (N=41) | 1.83 | 0.70 | 4.81 | 0.222 |
| Angpt2 (N=42) | 1.62 | 0.79 | 3.29 | 0.186 |
| BMP10 (N=42) | 0.83 | 0.44 | 1.56 | 0.560 |
| FGF23 (N=42) | 1.92 | 0.71 | 5.20 | 0.201 |
| IGFBP7 (N=42) | 0.90 | 0.48 | 1.69 | 0.738 |

**Supplement table 13 -** Cox regression of log-transformed biomarkers in relation to combined outcomes in patients with incident AF. Statistically significant regression models (p<0.05) are marked in bold. Analyses are based on 19 events. *Numbers in parentheses are cases included in the analysis. **Model adjusted for age, sex, systolic blood pressure, BMI, diabetes, dyslipidaemia, smoking and eGFR. Combined endpoint includes: all-cause mortality, stroke/TIA, myocardial infarction, coronary heart disease, heart failure. Abbreviations: Angpt2 – angiopoietin 2, BMP10 – bone morphogenetic protein 10, FGF23 – fibroblast growth factor 23, IGFBP7 - insulin-like growth factor binding protein 7, NTproBNP - N-terminal pro brain-natriuretic peptide

**For recurrent AF:**

| Biomarkers (N)* | HR | 95% Confidence interval | | p-value |
| --- | --- | --- | --- | --- |
|  |  | **lower** | **upper** |  |
| Multivariable adjustment** | | | | |
| NTproBNP (N=396) | 1.33 | 1.04 | 1.73 | **0.029** |
| Angpt2 (N=396) | 1.18 | 0.97 | 1.46 | **0.106** |
| BMP10 (N=395) | 1.26 | 1.01 | 1.58 | **0.034** |
| FGF23 (N=395) | 1.26 | 1.01 | 1.55 | **0.037** |
| IGFBP7 (N=395) | 1.24 | 0.96 | 1.63 | **0.094** |

**Supplement table 14** -Cox regressions for log-transformed biomarkers for recurrent AF in the high risk cohort. Note. Significant regressions (p <0.05) marked in bold. Analyses are based on 138 events. *Numbers in parentheses are cases included in the analysis. **Model adjusted for age, sex, systolic blood pressure, BMI, diabetes, dyslipidaemia, smoking and heart failure and eGFR. Abbreviations: Angpt2 – angiopoietin 2, BMP10 – bone morphogenetic protein 10, FGF23 – fibroblast growth factor 23, IGFBP7 - insulin-like growth factor binding protein 7, NTproBNP - N-terminal pro brain-natriuretic peptide

**For combined CVD outcomes in patients with AF at baseline:**

| Biomarkers (N)* | HR | 95% Confidence interval | | p-value |
| --- | --- | --- | --- | --- |
|  |  | **lower** | **upper** |  |
| Multivariable adjustment** | | | | |
| NTproBNP (N=508) | 1.22 | 0.89 | 1.57 | 0.275 |
| Angpt2 (N=508) | 1.01 | 0.77 | 1.17 | 0.697 |
| BMP10 (N=510) | 1.02 | 0.79 | 1.35 | 0.729 |
| FGF23 (N=510) | 1.14 | 0.96 | 1.40 | 0.099 |
| IGFBP7 (N=510) | 1.46 | 1.17 | 1.79 | **<0.001** |

**Supplement table 15 -** Cox regression for log-transformed biomarkers for the combined outcomes in patients with prevalent AF. Note. Significant regressions (p < 0.05) marked in bold. Analyses are based on 81 events *Numbers in parentheses are cases included in the analysis. **Model adjusted for age, sex, systolic blood pressure, BMI, diabetes, dyslipidaemia, smoking and heart failure. Combined endpoint includes: all-cause mortality, stroke/TIA, myocardial infarction, coronary heart disease, heart failure and eGFR. Abbreviations: Angpt2 – angiopoietin 2, BMP10 – bone morphogenetic protein 10, – fibroblast growth factor 23, IGFBP7 - insulin-like growth factor binding protein 7, NTproBNP - N-terminal pro brain-natriuretic peptide

**For all-cause mortality in patients with AF at baseline:**

| Biomarkers (N)* | HR | 95% Confidence interval | | p-value |
| --- | --- | --- | --- | --- |
|  |  | **lower** | **upper** |  |
| Multivariable adjustment** | | | | |
| NTproBNP (N=515) | 2.14 | 1.19 | 3.89 | **0.011** |
| Angpt2 (N=515) | 1.33 | 0.96 | 1.88 | 0.111 |
| BMP10 (N=517) | 1.00 | 0.71 | 1.68 | 0.674 |
| FGF23 (N=515) | 1.30 | 0.98 | 1.79 | 0.087 |
| IGFBP7 (N=517) | 1.59 | 1.09 | 2.23 | **0.011** |

**Supplement table 16** - Cox regressions adjusted for log-transformed biomarkers for all-cause mortality in patients with prevalent AF. Note. Significant regressions (p <0.05) marked in bold. Analyses are based on 27 events. *Numbers in parentheses are cases included in the analysis. **Model adjusted for age, sex, systolic blood pressure, BMI, diabetes, dyslipidaemia, smoking and heart failure and eGFR. Abbreviations: Angpt2 – angiopoietin 2, BMP10 – bone morphogenetic protein 10, FGF23 – fibroblast growth factor 23, IGFBP7 - insulin-like growth factor binding protein 7, NTproBNP - N-terminal pro brain-natriuretic peptide
